# Two parallel lateral hypothalamic GABAergic projections differentially modulate pain and anxiety in mice

**DOI:** 10.1371/journal.pbio.3004096

**Published:** 2026-11-05

**Authors:** Yi-Ling Sun, Yi-Ting Peng, Xue Zhang, Cui Yin, Cheng Xiao, Junping Chen, Chunyi Zhou

**Affiliations:** 1 Jiangsu Province Key Laboratory of Anesthesiology, School of Anesthesiology, Xuzhou Medical University, Xuzhou, Jiangsu, China; 2 Jiangsu Province Key Laboratory of Anesthesiology and Brain Science, Xuzhou Medical University, Xuzhou, Jiangsu, China; 3 Department of Anesthesiology, Ningbo No. 2 Hospital, Ningbo, Zhejiang, China; Columbia University Irving Medical Center, UNITED STATES OF AMERICA

## Abstract

The lateral hypothalamus (LH) is fundamentally involved in motivated behaviors, yet the precise functional segregation of its GABAergic projections in modulating pain and its affective dimensions remains poorly understood. Here, using male and female mice, we revealed that LH GABAergic neurons sent parallel projections to two pivotal pain and affect processing centers: the lateral parabrachial nucleus (LPB) and the paraventricular nucleus of the thalamus (PVT). In vivo fiber photometry revealed that LH GABAergic terminals in both the PVT and LPB are robustly activated by noxious, aversive, and rewarding stimuli, indicating their roles in encoding salient and affective information. Critically, we uncovered a remarkable functional segregation of these LH GABAergic pathways. Optogenetic activation of the LH-PVT projection exerted anxiogenic effects without altering pain sensitivity in both naïve and neuropathic pain mice; whereas optogenetic inhibition of this pathway exerted significant analgesic and anxiolytic effects specifically in neuropathic pain, highlighting a context-dependent modulation of pain and affect. In contrast, optogenetic activation of the LH-LPB projection induced contralateral pronociception without impacting affective behaviors, conversely, inhibition of this pathway significantly elevated contralateral pain thresholds in both physiological and neuropathic pain conditions. We also demonstrated that stimulation of the LH-LPB projection acted via a disinhibitory mechanism involving local LPB GABAergic interneurons to enhance activity of LPB glutamatergic neurons. Collectively, our findings delineate two parallel and functionally segregated LH GABAergic output pathways that differentially control distinct aspects of pain experience, thereby offering novel circuit-specific targets for the treatment of chronic pain and its debilitating emotional comorbidities.

## Abstract 2

Pain is intimately linked with emotional processing, but the neuronal circuitry delineating this relationship is incompletely understood. This study characterizes two parallel GABAergic outputs from the lateral hypothalamus that control these distinct aspects of pain experience.

## Introduction

Chronic pain, particularly neuropathic pain, affects millions globally, with a substantial 30%–50% of patients concurrently experiencing debilitating anxiety and depression [1,2]. This pervasive comorbidity underscores a fundamental yet incompletely understood interplay between nociceptive processing and emotional regulation [2,3]. Elucidating the specific neural circuits that bridge these sensory and affective domains is paramount, not only for deciphering the complex nature of chronic pain but also for developing targeted therapeutic strategies for its significant emotional comorbidities.

Among the brain regions crucially implicated in both nociception and affective processing, the lateral parabrachial nucleus (LPB) and the paraventricular nucleus of the thalamus (PVT) emerge as pivotal nodes. The LPB is a critical hub positioned at the confluence of peripheral sensory information and various affective behavioral states. This region receives direct nociceptive input from the dorsal horn of the spinal cord, and its neuronal populations are highly responsive to acute noxious stimuli [4,5]. Furthermore, sustained activation of excitatory LPB neurons has been demonstrated to induce a chronic pain-like state [6–8]. This strategic anatomical and functional position underscores the LPB’s significant role in pain processing and highlights its potential as a crucial site for pain modulation [9]. Concurrently, the PVT, an almost 100% glutamatergic midline thalamic nucleus, functions as a key integrator of stress, arousal, emotional and pain information, receiving diverse inputs and extensively projecting to limbic structures like the nucleus accumbens and prefrontal cortex [10–12]. Therefore, the dynamic contributions and coordinated involvement of both the LPB and PVT are essential for the holistic integration of sensory and affective components of pain experience.

The lateral hypothalamus (LH), traditionally recognized for its role in homeostatic behaviors, has recently garnered significant attention for its broader involvement in stress responses, reward, and multifaceted pain modulation [13–16]. Critically, the LH harbors diverse neuronal populations, including GABAergic neurons, which represent major inhibitory projection neurons [17]. These LH GABAergic neurons are uniquely positioned to exert widespread and potentially differentiated influence over various brain functions due to their extensive efferent projections to numerous subcortical and cortical regions, extending beyond their well-known targets. Notably, LH GABAergic neurons exhibit remarkable functional heterogeneity, mediating both reward-seeking behaviors (e.g., feeding, hunting, social interaction) [18,19] and avoidance responses [20], with specific subsets even encoding both punishment and reward signals [21]. This functional diversity allows them to flexibly modulate behavior based on internal state and external cues [22]. However, the specific contributions of distinct LH GABAergic projections to key pain- and affect-processing centers, particularly the LPB and PVT, and how these separate output pathways might differentially modulate pain perception versus pain-related negative affect, remain largely unexplored. Unraveling the functional roles of these specific LH GABAergic output pathways is essential for understanding the intricate interplay between hypothalamic regulation, ascending pain pathways, and the emotional processing of pain, potentially offering novel targets for intervention.

In this study, we employed a multi-faceted approach, combining optogenetic, chemogenetic, and fiber photometry techniques, to dissect the anatomical and functional heterogeneity of LH GABAergic projections specifically targeting the LPB and PVT. Our primary objective is to differentiate the unique contributions of these parallel LH GABAergic output pathways to the dimensions of pain perception versus pain-related negative affect, and to unravel their underlying circuit mechanisms, offering insights into hypothalamic regulation of pain perception and its affective dimensions.

## Results

### Identification and anatomical characterization of LH GABAergic projections to the PVT and LPB

To investigate the anatomical connectivity of LH GABAergic neurons to downstream regions involved in pain and emotion, specifically the PVT and LPB, we employed a retrograde tracing strategy combined with immunohistochemistry and electrophysiological recordings. To retrogradely label LH projection neurons, AAV retro-hSyn-mCherry and AAV retro-hSyn-eGFP were injected into the PVT and LPB, respectively, of wild-type (WT) mice (Fig 1A). Viral expression at the injection sites was confirmed in the PVT and LPB (Fig 1B), which resulted in significant retrograde labeling of neuron populations within the LH (Fig 1C). Subsequent immunofluorescence staining for GABA revealed that a substantial proportion of these retrogradely labeled neurons were GABAergic along the anteroposterior (AP) axis of the LH, demonstrating that LH GABAergic neurons project to both target areas (Fig 1D–1F). Quantitative analysis demonstrated that the labeling of PVT-projecting neurons was more prevalent than that of LPB-projecting neurons at the anterior levels of the LH, whereas their numbers were comparable in other AP planes (Fig 1G); both PVT- and LPB-projecting LH neurons accounted for over 30% of LH neurons labeled with GABA-antibody (Fig 1H). Among the total retrogradely labeled GABAergic neurons, the majority projected to either the PVT (55.4%) or the LPB (36.9%), with a small subset projecting to both regions (7.5%) (Fig 1I).

**Fig 1:**
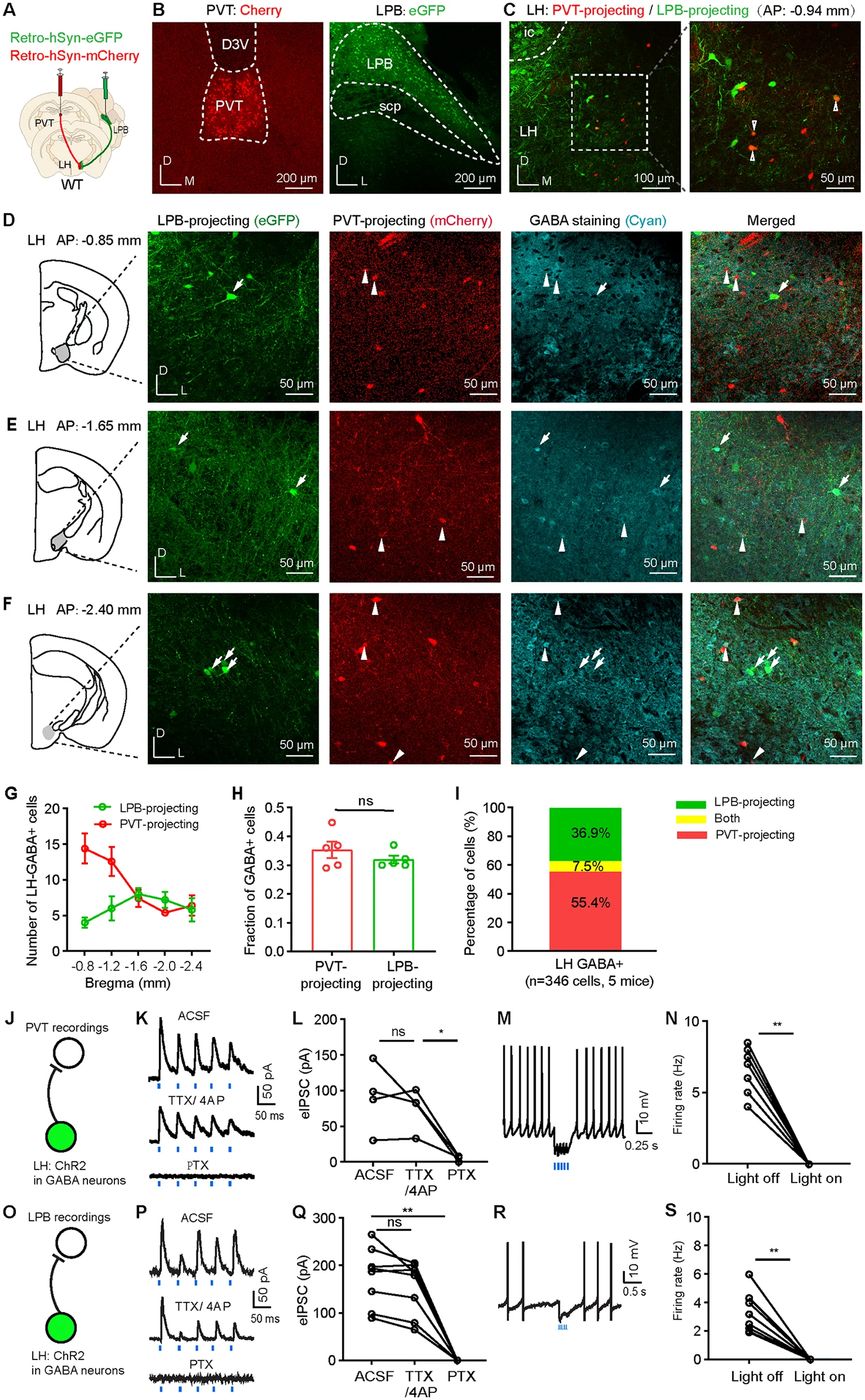
Anatomical and functional characterization of LH GABAergic projections to the PVT and LPB. **(A)** Schematic illustration of the retrograde tracing strategy. AAV retro-hSyn-eGFP and AAV retro-hSyn-mCherry were injected into the LPB and PVT, respectively, in wild-type (WT) mice to retrogradely label neurons in the LH that project to these regions. **(B)** Representative images showing viral expression in the PVT (left) and LPB (right). scp, superior cerebellar peduncle. **(C)** Representative retrogradely labeled cells in the LH originating from the PVT and LPB. Open arrowheads indicate double-labeled neurons (mCherry+/eGFP+) in the LH that project to both the PVT and LPB. **(D–F)** Representative images and corresponding brain schematics illustrating the distribution of mCherry-positive (red, PVT-projecting) and eGFP-positive (green, LPB-projecting) neurons co-localized with GABA immunostaining (cyan) at different anteroposterior (AP) levels of the LH. White arrows indicate LPB-projecting neurons (eGFP+); white arrowheads indicate PVT-projecting neurons (mCherry+). **(G)** Number of LH-GABA+ neurons projecting to the LPB (green) and PVT (red) at indicated AP levels. **(H)** The fraction of GABA+ neurons among the retrogradely labeled population projecting to the PVT and LPB (*t* = 1.07, *P* = 0.32, *n* = 5 mice, unpaired *t* test). **(I)** Composition of the LH-GABAergic projection population. Percentage of neurons projecting to the LPB, PVT, or both (*n* = 346 neurons from 5 mice). **(J)** Schematic diagram of electrophysiological recordings from PVT neurons in brain slices upon blue light illumination of projections from LH GABAergic neurons transfected with ChR2-eYFP. **(K)** Representative traces of blue light-evoked IPSCs in a PVT neuron in artificial cerebrospinal fluid (ACSF), in the presence of TTX (tetrodotoxin) and 4-AP (4-aminopyridine), and after application of the GABA_A_ receptor antagonist picrotoxin (PTX, 50 μM). **(L)** Quantification of light-evoked IPSC amplitude in PVT neurons under different conditions (*F*_(2, 6)_ = 10.93, *P* = 0.01; *n* = 4 cells, RM one-way ANOVA). **(M)** Representative trace under current-clamp recording from a PVT neuron showing action potential firing inhibited by blue light stimulation. **(N)** Quantification of PVT neuron firing rate with and without optogenetic stimulation of the LH-PVT GABAergic projection (*t* = 12.49, *P* < 0.0001; *n* = 9 cells). **(O)** Schematic diagram of electrophysiological recordings from LPB neurons in brain slices upon blue light illumination of projections from LH GABAergic neurons transfected with ChR2-eYFP. **(P)** Representative traces of light-evoked IPSCs in an LPB neuron showing recordings in ACSF, TTX/4-AP, and after PTX. **(Q)** Quantification of light-evoked IPSC amplitude in LPB neurons in different conditions (*F*_(1.29, 9.02)_ = 58.00, *P* < 0.0001; *n* = 8 cells, one-way repeated measures ANOVA). **(R)** Representative trace of optogenetic inhibition of LPB neuron firing. **(S)** Quantification of LPB neuron firing rate with and without optogenetic stimulation of the LH-LPB GABAergic projection (*t* = 6.44, *P* = 0.0004; *n* = 8 cells, Paired *t t*es*t*). Data are presented as mean ± SEM. ns: not significant. * *P* < 0.05, ** *P* < 0.01. Blue light pulses (473 nm, 5 ms) are indicated by the blue bars in panels (K, M, P and **R)**. Orientation arrows: D, dorsal; M, medial; L, lateral. Data are available in S1 Data as a part of Supporting information.

Furthermore, we mapped the distribution of LH GABAergic fibers along the AP axis of the PVT and LPB by expressing ChR2-eYFP in LH GABAergic neurons in Vgat-cre mice (S1A–S1C and S1E Fig). Fluorescence intensity analysis revealed that the projection density was relatively consistent across the anterior and posterior PVT (S1D Fig). In contrast, the density of LH GABAergic fibers in the LPB showed a significant AP gradient with higher density in the anterior subregions compared to the posterior subregions (S1F Fig). These results indicate that the LH-LPB projection, but not the LH-PVT projection, exhibits distinct subregional patterns.

Functionally, we confirmed the inhibitory nature of these LH GABAergic projections to both the PVT and LPB using optogenetics coupled with whole-cell patch-clamp recording. Optogenetic stimulation of LH GABAergic terminals in acute brain slices consistently evoked fast, monosynaptic inhibitory postsynaptic currents (IPSCs) in both PVT neurons (Fig 1J–1L) and LPB neurons (Fig 1O–1Q). These IPSCs were not sensitive to tetrodotoxin (TTX, 0.3 µM) and 4-aminopyridine (4-AP, 100 µM), indicating they were mediated by monosynaptic connections; they were abolished by picrotoxin (PTX, 50 µM), confirming their GABAergic identity (Fig 1K, 1L, 1P, and 1Q). Furthermore, optogenetic activation of these projections significantly inhibited the spontaneous firing of both PVT neurons (Fig 1M and 1N) and LPB neurons (Fig 1R and 1S).

Collectively, these data establish that specific populations of LH GABAergic neurons form functional inhibitory synapses onto neurons within both the PVT and LPB.

### The LH-PVT GABAergic projection differentially modulates pain and anxiety-like behaviors in neuropathic pain

To investigate the role of the LH-PVT GABAergic projection in modulating neuropathic pain and associated affective behaviors, we first established the neuropathic pain model and performed optogenetic manipulation of this pathway. AAVs expressing ChR2-eYFP (for activation), NpHR-eYFP (for inhibition), or eYFP (as control) were unilaterally injected into the LH of Vgat-Cre mice to achieve selective expression in LH GABAergic neurons (Fig 2A). Optical fibers were then implanted above the PVT to deliver light stimulation to these projections (Fig 2A). Fluorescence imaging confirmed the successful expression of ChR2-eYFP (Fig 2B) and NpHR-eYFP (Fig 2C) in LH GABAergic neurons and their projections to the PVT.

**Fig 2:**
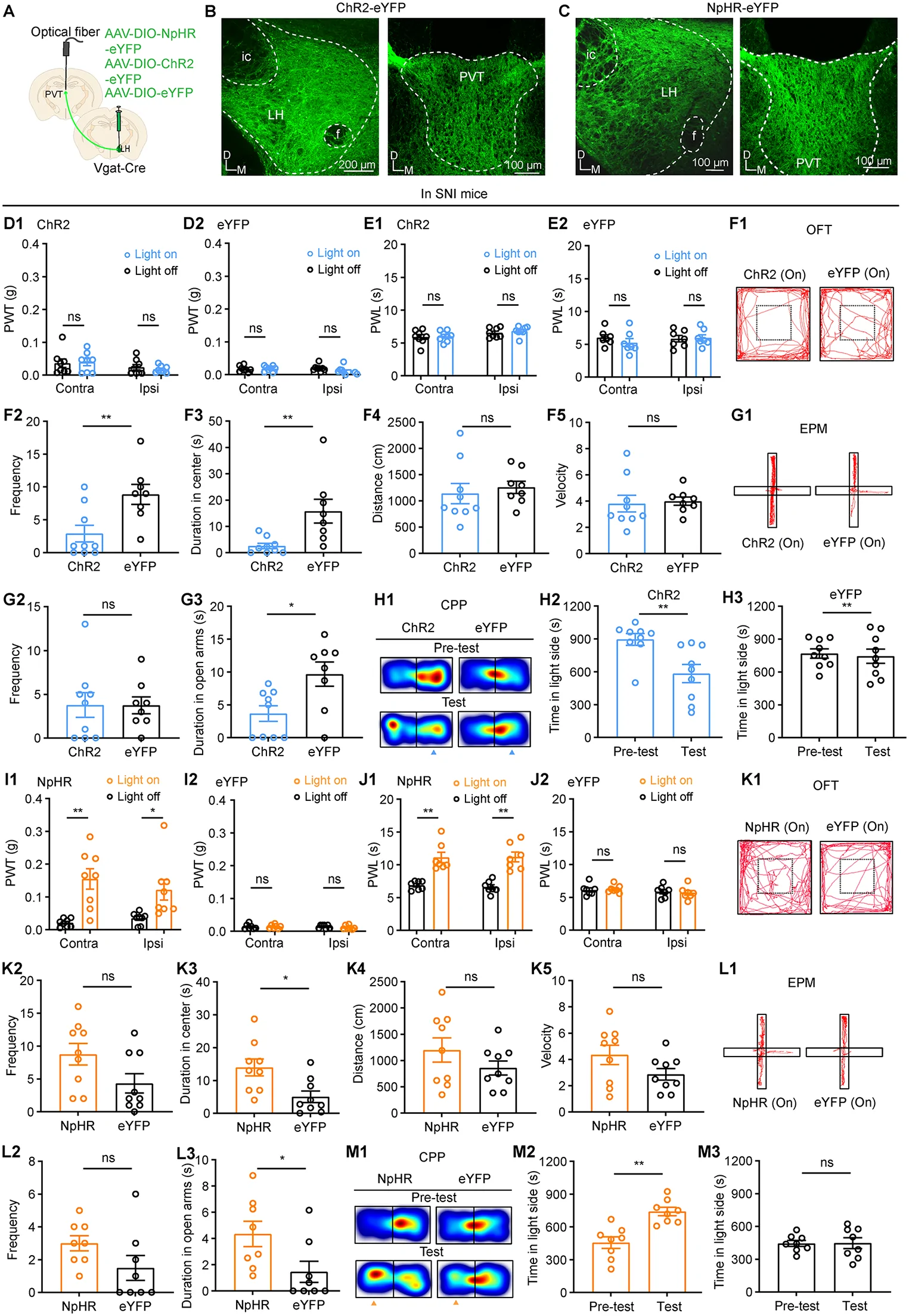
Optogenetic manipulation of the LH-PVT GABAergic projection modulates pain perception and affective behaviors. **(A)** Schematic illustration of virus injection and optical fiber implantation in Vgat-Cre mice. AAV-EF1α-DIO-ChR2-eYFP or AAV-EF1α-DIO-NpHR-eYFP or AAV-EF1α-DIO-eYFP was injected into the LH and an optical fiber was implanted above the paraventricular nucleus of the thalamus (PVT) for photostimulation. **(B, C)** Representative images showing the expression of ChR2-eYFP (B) or NpHR-eYFP (C) in LH GABAergic neurons and their terminals in the PVT (right panels). An optical fiber is implanted above the PVT. **(D1–D2)** Mechanical paw withdrawal threshold (PWT) in ChR2 (D1, *F*_(1, 14)_ = 0.044, *P* = 0.84; *n* = 8 per group) and eYFP (D2, *F*_(1, 14)_ = 0.12, *P* = 0.73; *n* = 8 per group) mice before and during blue light stimulation. **(E1–E2)** Thermal paw withdrawal latency (PWL) in ChR2 (E1, *F*_(1, 14)_ = 0.88, *P* = 0.37; *n* = 8 per group) and eYFP (E2, *F*_(1, 12)_ = 0.41, *P* = 0.53; *n* = 7 per group) mice before and during blue light stimulation. **(F1–F5)** Representative traces and quantitative analysis of open field test (OFT) behaviors for ChR2 and eYFP mice during blue light stimulation (F2: *t* = 3.03, *P* = 0.008; F3: *t* = 3.02, *P* = 0.009; F4: *t* = 0.51, *P* = 0.62; F5: *t* = 0.25, *P* = 0.80; *n* = 9 in ChR2, *n* = 8 in eYFP). **(G1–G3)** Representative traces and quantitative analysis of elevated plus maze (EPM) behaviors for ChR2 and eYFP mice during blue light stimulation (G2: *t* = 0.016, *P* = 0.99; G3: *t* = 2.81, *P* = 0.013; *n* = 9 in ChR2, *n* = 8 in eYFP). **(H1)** Representative heatmaps of conditioned place preference (CPP) test for ChR2 and eYFP mice before (Pre-test) and after (Test) conditioning. Blue triangles indicate the light-paired chambers. During the 3-day conditioning phase, mice were restricted to the non-paired chamber for 30 min in the morning and to the light-paired chamber for 30 min in the afternoon, during which they received optogenetic stimulation (473 nm, 20 Hz, 5-ms pulse width). **(H2, H3)** Quantitative analysis of CPP (H2: *t* = 4.92, *P* = 0.0012; H3: *t* = 0.47, *P* = 0.65; *n* = 9 per group). **(I1, I2)** PWT in NpHR (I1, *F*_(1, 14)_ = 27.85, *P* = 0.0001; *n* = 8 per group) and eYFP (I2, *F*_(1, 12)_ = 0.46, *P* = 0.51; *n* = 7 per group) mice before and during yellow light stimulation. **(J1, J2)** PWL in NpHR (J1, *F*_(1, 12)_ = 67.04, *P* < 0.0001; *n* = 7 per group) and eYFP (J2, *F*_(1, 12)_ = 0.0078, *P* = 0.93; *n* = 7 per group) mice before and during yellow light stimulation. **(K1–K5)** Representative traces and quantitative analysis of OFT behaviors for NpHR and eYFP mice during yellow light stimulation (K2: *t* = 2.006, *P* = 0.062; K3: *t* = 2.86, *P* = 0.011; K4: *t* = 1.28, *P* = 0.22; K5: *t* = 1.73, *P* = 0.10; *n* = 9 per group). **(L1–L3)** Represen*t*ative traces and quantitation of EPM behaviors for NpHR and eYFP mice during yellow light stimulation (L2: *t* = 1.69, *P* = 0.11; L3: *t* = 2.30, *P* = 0.038; *n* = 8 per group). **(M1)** Representative heatmaps of CPP test for NpHR and eYFP mice before (Pre-test) and after (Test) conditioning. The conditioning paradigm was similar to Fig 2H1, except that 589 nm yellow light (constant) was used for inhibition. **(M2, M3)** Quantitative analysis of CPP (M2: *t* = 6.54, *P* = 0.0003; M3: *t* = 0.058, *P* = 0.96; *n* = 8 per group). Da*t*a are presented as mean ± SEM. *P < 0.05, **P < 0.01. ns: not significant. Contra: contralateral; Ipsi: Ipsilateral. Two-way ANOVAs were used for panels (D1, D2, E1, E2, I1, I2, J1, J2), followed by Tukey’s post-hoc tests where appropriate. Unpaired *t*-tests were used for panels (F2–F5, G2, G3, K2–K5, L2, L3). Paired *t*-tests were used for panels (H2, H3, M2, M3). Orien*t*ation arrows: D, dorsal; M, medial. Data are available in S1 Data as a part of Supporting information.

Following these surgical procedures, mice underwent spared nerve injury of the sciatic nerve (SNI) or sham surgery. Specifically, SNI was performed on either contralateral or ipsilateral hind paws relative to the unilateral viral injection site. Four weeks after surgery, these SNI mice developed consistent mechanical allodynia and thermal hyperalgesia, evidenced by significantly reduced paw withdrawal threshold (PWT) and paw withdrawal latency (PWL), respectively (S2A and S2B Fig). Furthermore, these neuropathic pain mice exhibited anxiety-like behaviors 4 weeks after SNI surgery, characterized by decreased time and frequency in the open arms of the elevated plus maze (EPM) (S2C and S2D Fig) and reduced frequency of entries into and duration in the center area in the open field test (OFT), without affecting locomotor activity (S2E–S2H Fig).

Behavioral assessments were performed under unilateral blue light stimulation. We found that acute activation of the LH-PVT GABAergic projection did not significantly alter pain sensitivity, as evidenced by unchanged mechanical PWT and thermal PWL in ChR2 mice compared to eYFP controls (Fig 2D1, 2D2, 2E1, and 2E2). However, this activation significantly altered affective behaviors. Specifically, ChR2 mice showed decreased frequency of entries into and duration in the center area of the OFT (Fig 2F1–2F3), and decreased duration in the open arms of the EPM (Fig 2G1–2G3), compared to eYFP controls, suggesting an anxiogenic-like effect. Locomotor activity, as indicated by total distance and velocity in the OFT, remained unchanged (Fig 2F4 and 2F5). Furthermore, in the conditioned place preference (CPP) test, ChR2 mice, but not eYFP mice, spent significantly less time in the light-paired chamber during the test phase compared to their pre-test baseline (Fig 2H1–2H3). These results indicate that stimulation of the LH-PVT projection caused aversion. This anxiogenic effect of the LH-PVT projection is also observed in naïve mice (S3A1–S3E3 Fig).

We next investigated the effects of optogenetic inhibition on pain and anxiety-like behaviors in neuropathic pain mice. Yellow light stimulation in NpHR mice resulted in a significant increase in mechanical PWT (Fig 2I1 and 2I2) and thermal PWL (Fig 2J1 and 2J2) in both contralateral and ipsilateral SNI-injured hind paws, demonstrating analgesic effects compared to eYFP controls or light-off conditions. In terms of affective behaviors, NpHR mice displayed an increased duration in the center area of the OFT (Fig 2K1–2K3) and an increased duration in the open arms of the EPM, compared to eYFP controls (Fig 2L1–2L3), suggesting anxiolytic-like effect. Locomotor activity was not affected (Fig 2K4 and 2K5). In the CPP test, NpHR mice spent significantly longer time in the light-paired chamber during the test phase compared to their pre-test baseline (Fig 2M1–2M3), indicating a strong preference for the light stimulation, consistent with the induction of positive affective states. Notably, this dual analgesic and anxiolytic effect is selectively recruited during neuropathic pain, as inhibition of the LH-PVT projection showed no significant behavioral changes in naïve animals (S3F1–S3M Fig).

Collectively, these results strongly suggest that activation of the LH-PVT GABAergic projection increases anxiety and induces negative affective states, while its inhibition provides analgesic effects, reduces anxiety, and promotes positive affective states. This highlights the LH-PVT GABAergic circuit as an important component in the neural circuitry of pain and affect.

### The LH-LPB GABAergic projection bidirectionally regulates nociception across physiological and neuropathic pain states

We next investigated the functional roles of another projection from the LH: the LH-LPB GABAergic pathway. For optogenetic activation experiments, AAVs expressing ChR2-eYFP or eYFP as control were unilaterally injected into the LH of Vgat-Cre mice to selectively target LH GABAergic neurons. Optical fibers were then implanted above the ipsilateral LPB to deliver light stimulation to this projection (Figs 3A, S4A, and S4B). As confirmed by fluorescence imaging, successful expression of ChR2-eYFP was observed in LH GABAergic neurons with abundant projections reaching the LPB (Fig 3B).

**Fig 3:**
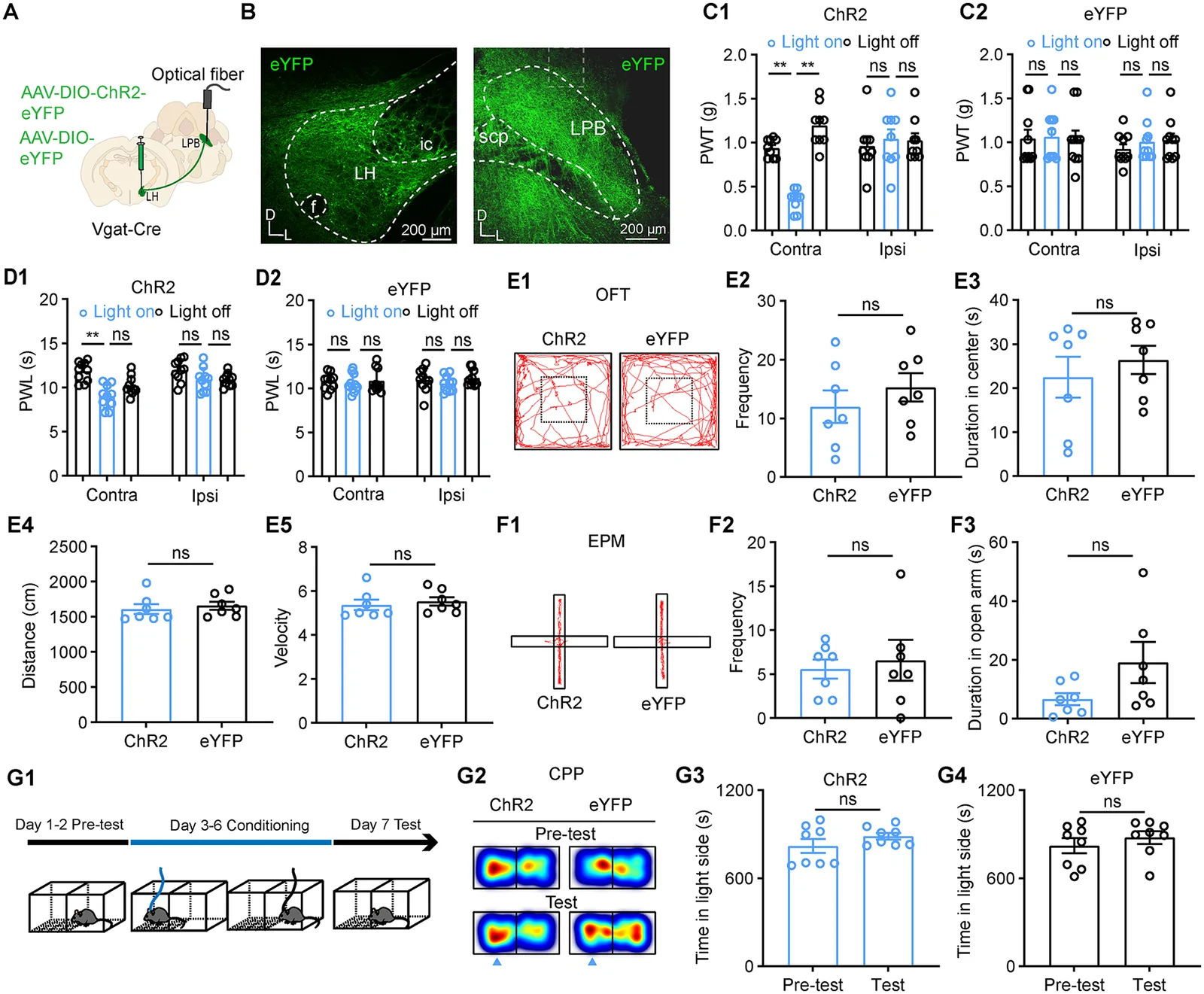
Optogenetic activation of the LH-LPB pathway induces pronociception but not anxiety-like behavior in naive mice. **(A)** Schematic diagram illustrating AAV-EF1α-DIO-ChR2-eYFP or AAV-EF1α-DIO-eYFP injection into the LH and optical fiber implantation above the LPB in Vgat-Cre mice. **(B)** Representative coronal brain sections showing viral expression of ChR2-eYFP (green fluorescence) in the LH (left) and eYFP-positive axon terminals in the LPB (right). **(C1, C2)** Mechanical PWT in ChR2 (C1) and eYFP (C2) groups during light on (blue) and light off (black) conditions for contralateral and ipsilateral (Ipsi) hind paws (C1, *F*_(1.79, 28.63)_ = 13.56, *P* = 0.0001; *n* = 9 per group; C2, *F*_(1.78, 31.97)_ = 0.53, *P* = 0.57; *n* = 10 per group). **(D1, D2)** Thermal PWL in ChR2 (D1) and eYFP (D2) mice during light on (blue) and light off (black) conditions for contralateral and ipsilateral hind paws (D1, *F*_(1.78, 31.94)_ = 13.92, *P* < 0.0001; *n* = 10 per group; D2, *F*_(1.80, 32.32)_ = 1.17, *P* = 0.32; *n* = 10 per group). **(E1–E5)** Representative traces and quantitative analysis of OFT behaviors for ChR2 and eYFP upon blue light stimulation (E2, *t* = 0.89, *P* = 0.39; E3, *t* = 0.70, *P* = 0.50; E4, *t* = 0.52, *P* = 0.60; E5, *t* = 0.52, *P* = 0.60; *n* = 7 per group). **(F1–F3)** Represen*t*ative traces and quantitative analysis of EPM behaviors for ChR2 and eYFP upon blue light stimulation (F2, *t* = 0.39, *P* = 0.70; F3, *t* = 1.72, *P* = 0.11; *n* = 7 per group). **(G1)** The experimen*t*al timeline for CPP test. The conditioning paradigm was performed as described in Fig. 2H1. **(G2)** Representative heatmaps showing tracks from a ChR2 mouse and an eYFP mouse in the CPP chamber during pre-test and test phases. **(G3, G4)** No significant change in time spent in the light side during pre-test or test phases in ChR2 (G3, *t* = 1.26, *P* = 0.25; *n* = 8 in ChR2) or eYFP (G4, *t* = 0.68, *P* = 0.52; *n* = 8 in eYFP) mice. Da*t*a are presented as mean ± SEM. * *P* < 0.05; ** *P* < 0.01. Two-way ANOVAs with Tukey’s post hoc tests were used in panels C1, C2, D1, D2. Two-tailed unpaired Student’s *t*-tests were used in panels E2–E5, F2, F3. Two-*t*ailed paired *t* test were used for panels G3-G4. Orien*t*ation arrows: D, dorsal; L, lateral. Data are available in S1 Data as a part of Supporting information.

Acute activation of the LH-LPB projection robustly induced contralateral pronociception in naive mice. This was evidenced by significantly reduced mechanical PWT and thermal PWL on the contralateral, but not ipsilateral hind paws (Fig 3C1–3D2), suggesting that activation of this pathway exerts a potent pronociceptive effect with contralaterality. Importantly, activation of the LH-LPB projection did not significantly alter anxiety-like behaviors in the OFT (Fig 3E1–3E5) and the EPM (Fig 3F1–3F3) or CPP in naive mice (Fig 3G1–3G4), indicating a specific role in nociception rather than general affective states. These findings led us to hypothesize that overactivity of the LH-LPB pathway might significantly contribute to neuropathic pain states, and that its inhibition could offer therapeutic benefits.

Given the contralateral-specific pronociceptive effect observed with activation of the LH-LPB GABAergic projection in naive mice, we next investigated the therapeutic potential of inhibiting this pathway in neuropathic pain, focusing our behavioral assessment on the contralateral hind paw. In these experiments, AAV-EF1α-DIO-NpHR-eYFP or AAV-EF1α-DIO-eYFP was unilaterally injected into the LH, and optical fibers were implanted above the ipsilateral LPB (Figs 4A, 4B, and S4C). SNI was induced on the contralateral hind paw relative to the virus injection and optical fiber implantation, with behavioral testing specifically focused on the SNI-affected hind paw. We observed that unilateral yellow light illumination of the LH-LPB GABAergic projection in NpHR mice significantly alleviated neuropathic pain, as evidenced by a substantial increase in mechanical PWT (Fig 4C1) and thermal PWL (Fig 4C2) on the contralateral SNI-injured hind paw compared to eYFP controls or light-off conditions. In contrast, this inhibition failed to alter anxiety-like behaviors (Fig 4D1–4E3) or induce preference in the CPP (Fig 4F1–4F3).

**Fig 4:**
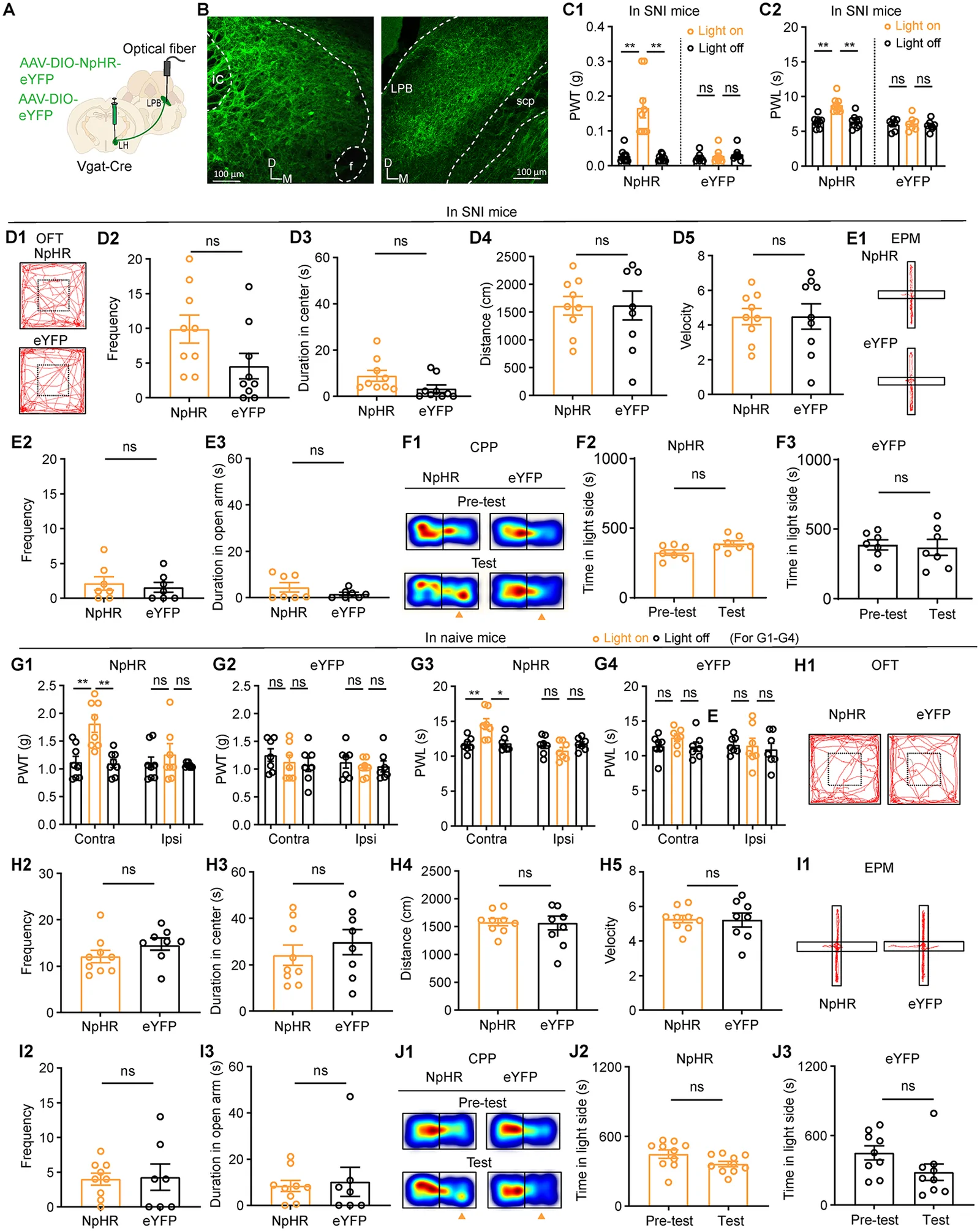
Optogenetic inhibition of the LH-LPB pathway alleviates neuropathic pain and increases physiological pain thresholds without affecting affective behaviors. **(A, B)** Schematic of viral injection and fiber implantation **(A)**, and representative images of NpHR-eYFP expression in LH neurons and terminals in the LPB **(B)**. **(C1, C2)** Mechanical PWT and (C1) thermal PWL (C2) in SNI mice during yellow light on (orange) and light off (black) conditions (C1: *F*_(1.04, 14.50)_ = 17.22, *P* = 0.0008; *n* = 9 in NpHR, *n* = 7 in eYFP; C2: *F*_(1.37, 19.13)_ = 25.38, *P* < 0.0001, *n* = 9 in NpHR, *n* = 7 in eYFP). **(D1–D5)** Representative traces and quantitative analysis of OFT behaviors in SNI mice during yellow light stimulation (D2: *t* = 1.95, *P* = 0.069; D3: *t* = 1.95, *P* = 0.068; D4: *t* = 0.015, *P* = 0.99; D5: *t* = 0.015, *P* = 0.99; *n* = 9 per group). **(E1–E3)** EPM resul*t*s for NpHR and eYFP mice. (E1-E3) Representative traces and quantitative analysis of EPM behaviors in SNI mice during yellow light stimulation (E2: *t* = 0.48, *P* = 0.64; E3: *t* = 1.34, *P* = 0.21; *n* = 7 per group). **(F1–F3)** Represen*t*ative heatmaps (F1) and quantitative analysis of CPP in SNI mice (F2: *t* = 1.93, *P* = 0.10, *n* = 7; F3: *t* = 0.25, *P* = 0.81, *n* = 7). The condi*t*ioning paradigm was performed as described in Fig 2M1. **(G1–G4)** Mechanical PWT (G1, G2) and thermal PWL (G3, G4) in NpHR and eYFP mice during light on (yellow) and light off (black) conditions for contralateral and ipsilateral paws in naive mice (G1: *F*_(1.66, 23.22)_ = 13.04, *P* = 0.0003; G2: *F*_(1.55, 18.64)_ = 1.16; *n* = 8 per group; G3: *F*_(1.82, 21.83)_ = 3.58, *P* = 0.049; G4: *F*_(1.85, 22.14)_ = 1.01, *P* = 0.37; *n* = 7 per group). **(H1–H5)** Representative traces and quantitative analysis of OFT behaviors in naive mice (H2: *t* = 1.28, *P* = 0.22; H3: *t* = 0.82, *P* = 0.42; H4: *t* = 0.11, *P* = 0.92; H5: *t* = 0.11, *P* = 0.91; *n* = 8-9 per group). **(I1–I3)** Represen*t*ative traces and quantitative analysis of EPM behaviors in naive mice (I2: *t* = 0.15, *P* = 0.89; I3: *t* = 0.28, *P* = 0.78; *n* = 7-9 per group). **(J1–J3)** Represen*t*ative heatmaps and quantitative analysis of CPP in naive mice (J2: *t* = 2.02, *P* = 0.074, *n* = 10; J3: *t* = 1.85, *P* = 0.10, *n* = 9). Da*t*a are presented as mean ± SEM. * *P* < 0.05; ** *P* < 0.01. Contra: contralateral; Ipsi: Ipsilateral. Statistical analysis: Two-way ANOVAs with Tukey’s post hoc tests were used in panels (C1, C2, G1–G4). Two-tailed unpaired Student’s *t*-tests were used in panels (D2–D5, E2, E3, H2–H5, I2, I3). Two-*t*ailed paired *t*-tests were used for panels (F2, F3, J2, J3) *t*o compare pre-test vs. test within the same group. Orientation arrows: D, dorsal; M, medial. Data are available in S1 Data as a part of Supporting information.

Similarly, in naive mice, inhibiting this projection increased basal mechanical and thermal thresholds specifically on the contralateral paw (Fig 4G1–4G4), without affecting affective states or locomotor activity (Fig 4H1–4J3). Collectively, these results demonstrate that the LH-LPB GABAergic pathway selectively and unilaterally modulates nociception across both physiological and pathological states, distinct from the LH-PVT circuit’s role in affect.

### LH GABAergic terminal activity in the PVT and LPB encodes salient and/or affective stimuli

To further elucidate the underlying neuronal mechanisms by which LH GABAergic projections to the PVT and LPB modulate nociceptive and affective behaviors, we next performed in vivo fiber photometry to record calcium transients from these projection-specific terminals in freely moving mice. We specifically investigated how the activity of LH GABAergic terminals in both the PVT and LPB responds to various noxious, aversive, and appetitive stimuli.

For recordings from LH GABAergic terminals in the PVT, AAV-EF1α-DIO-GCaMP6s or AAV-EF1α-DIO-eYFP was stereotaxically injected into the LH of Vgat-Cre mice, and an optical fiber was implanted above the PVT (Figs 5A, S4D, and S4E). As confirmed by histological analysis, robust GCaMP6s expression was observed in LH GABAergic neurons and their terminals in the PVT (Fig 5B). We then examined calcium transients in these terminals in response to various stimuli. Mechanical noxious stimulation (2.0 g von Frey filament) on contralateral hind paw evoked a significant increase in GCaMP6 fluorescence in LH-PVT terminals compared to eYFP controls (Fig 5C1 and 5C2). Similarly, thermal noxious stimulation (48°C) also elicited a significant increase in GCaMP6 signal in LH-PVT terminals (Fig 5D1 and 5D2). Furthermore, aversive stimuli such as water spray to the face and exploration of the open arms in the EPM, both known to induce stress or aversion, significantly increased the activity of LH-PVT terminals (Fig 5E1 and 5E2 for water spray; Fig 5F1 and 5F2 for exploration of the open arms in the EPM). Importantly, a rewarding stimulus like sucrose licking also induced a significant increase in GCaMP6 fluorescence in LH-PVT terminals (Fig 5G1 and 5G2). These data suggest a broader encoding role of LH-PVT terminals in salient and/or appetitive events in addition to noxious and aversive stimuli.

**Fig 5:**
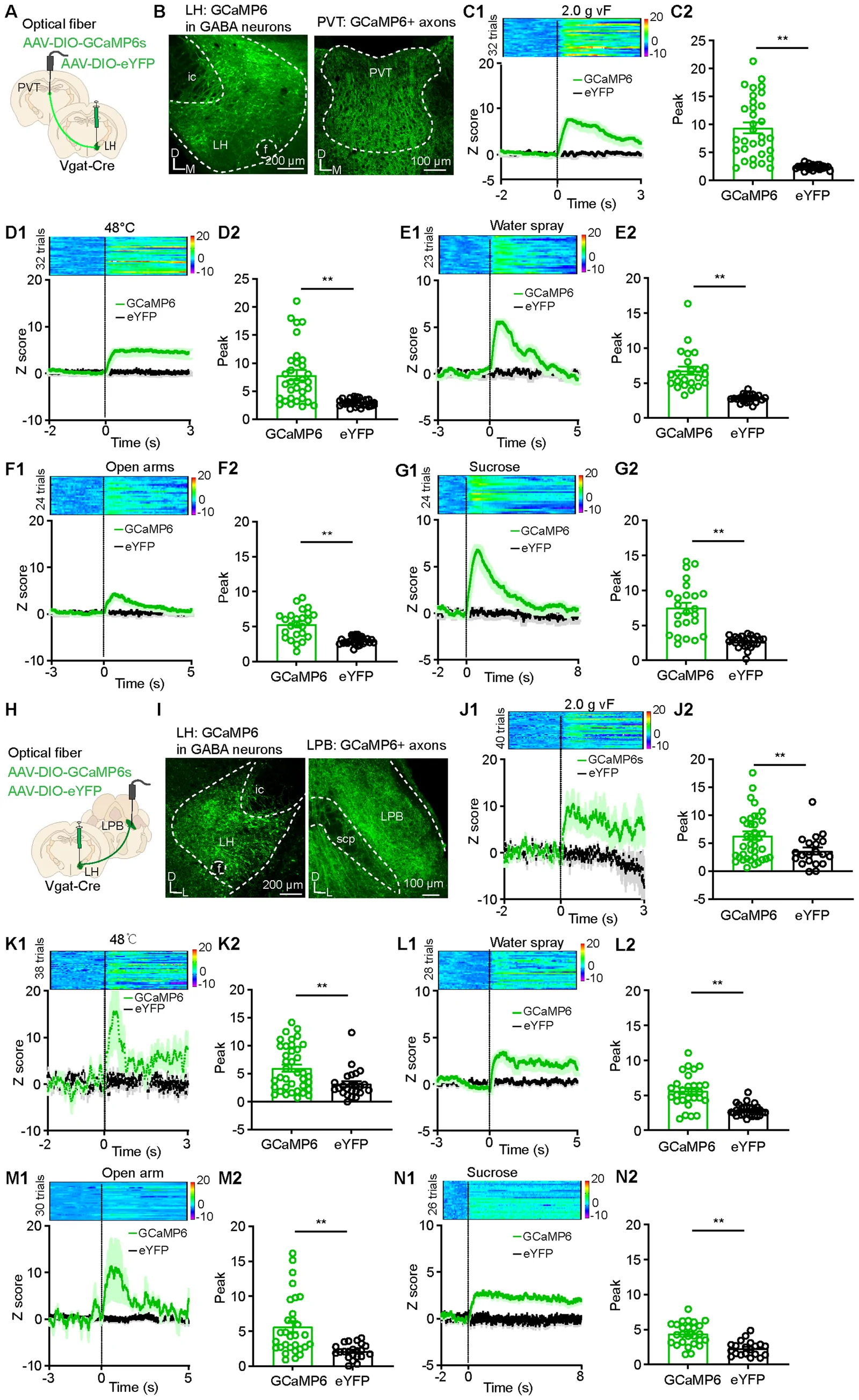
LH GABAergic terminal activity in the PVT and LPB encodes noxious and aversive stimuli. **(A)** Schematic of experimental setup for recording calcium transients from LH GABAergic terminals in the PVT. **(B)** Representative fluorescence images showing GCaMP6 expression in LH GABA neurons (left) and their axon fibers and terminals in the PVT (right). **(C1–D2)** Heatmaps and average Z-scores of GCaMP6s signals in LH-PVT terminals in response to mechanical (2.0 g vF; C1, C2) and thermal (48 °C; D1, D2) noxious stimuli (C2: *t* = 6.81, *P* < 0.0001; D2: *t* = 5.26, *P* < 0.0001; *n* = 28-32 *t*rials per group). **(E1–G2)** LH-PVT terminal activity in response to aversive water spray (E1, E2), exploration of EPM open arms (F1, F2), and rewarding sucrose licking (G1, G2) (E2: *t* = 6.46, *P* < 0.0001; F2: *t* = 6.06, *P* < 0.0001; G2: *t* = 6.41, *P* < 0.0001; *n* = 23-28 trials per group). **(H)** Schema*t*ic of experimental se*t*up for recording calcium transients from LH GABAergic terminals in the LPB. **(I)** Representative fluorescence images showing GCaMP6 expression in LH GABA neurons (left) and their axonal fibers and terminals in the LPB (right). **(J1–N2)** Fluorescence responses of GCaMP6 and eYFP signals to 2.0 g von Frey stimulation (J1, J2), 48 °C thermal stimulation (K1, K2), water spray on the cheek (L1, L2), exploration of the open arms in the EPM (M1, M2) and sucrose licking (N1, N2) (J2: *t* = 2.36, *P* = 0.022; *n* = 40 in GCaMP6s, *n* = 20 in eYFP; K2: *t* = 3.11, *P* = 0.0029; *n* = 38 in GCaMP6s, *n* = 24 in eYFP; L2: *t* = 5.94, *P* < 0.0001; *n* = 28 in GCaMP6s, *n* = 29 in eYFP; M2: *t* = 3.78, *P* = 0.0004; *n* = 30 in GCaMP6s, *n* = 22 in eYFP; N2: *t* = 4.40, *P* < 0.0001; *n* = 26 in GCaMP6s, *n* = 21 in eYFP). Da*t*a are presen*t*ed as mean ± SEM. * *P* < 0.05; ** *P* < 0.01. Two-*t*ailed unpaired Studen*t*’s *t*-tests were used in panels (C2, D2, E2, F2, G2, J2, K2, L2, M2, N2). The vertical dashed line indicates the onset of stimulation. Orien*t*ation arrows: D, dorsal; M, medial.Data are available in S1 Data as a part of Supporting information.

Next, we investigated the activity of LH GABAergic terminals in the LPB. The experimental setup included injection of AAV-EF1α-DIO-GCaMP6s or AAV-EF1α-DIO-eYFP into the LH and implantation of an optical fiber above the LPB in Vgat-Cre mice (Figs 5H; S4F, and S4G). Successful virus expression was verified histologically (Fig 5I). Consistent with our findings in the PVT, LH-LPB GABAergic terminals showed significant activation in response to mechanical noxious stimulation (2.0 g von Frey filament; Fig 5J1 and 5J2), thermal noxious stimulation (48 °C; Fig 5K1 and 5K2), aversive water spray (Fig 5L1 and 5L2), exploration of the open arms in the EPM (Fig 5M1, 5M2), and sucrose licking (Fig 5N1 and 5N2).

In summary, these fiber photometry results demonstrate that LH GABAergic projections to both the PVT and LPB are robustly activated by noxious, aversive, and rewarding stimuli. This suggests that both pathways play a crucial role in encoding salient environmental cues, including those associated with negative affective states, nociception, and appetitive experiences.

### Characterization of LH GABAergic projections to the LPB and their functional impact on LPB neuronal activity

We next focused our investigation on the LPB microcircuitry. The LPB, unlike the PVT, is a heterogeneous nucleus containing both glutamatergic projection neurons and GABAergic interneurons. These diverse neuronal populations critically shape the LPB’s output and can exert opposing effects on pain and aversion. Therefore, we sought to characterize the specific cellular targets of LH GABAergic projections and functional modulation of LPB excitatory and inhibitory neurons.

To elucidate the direct cellular targets of LH GABAergic projections within the LPB, we employed an anterograde *trans-*synaptic labeling strategy. Specifically, we injected AAV-hSyn-DIO-WGA-Flp into the LH of Vgat-Cre mice (Fig 6A). This virus expresses a WGA-Flp fusion protein in LH-GABAergic neurons, which is then transported *trans-*synaptically to label postsynaptic neurons. Concurrently, a Flp-dependent reporter (AAV-hSyn-fDIO-eYFP) was injected into the LPB to selectively label LPB neurons receiving direct synaptic inputs from LH GABAergic neurons. To determine the identity of these neurons, AAV-GAD67-mCherry was co-injected to label GABAergic cells, and Vglut2-antibody staining was used to label glutamatergic neurons.

**Fig 6:**
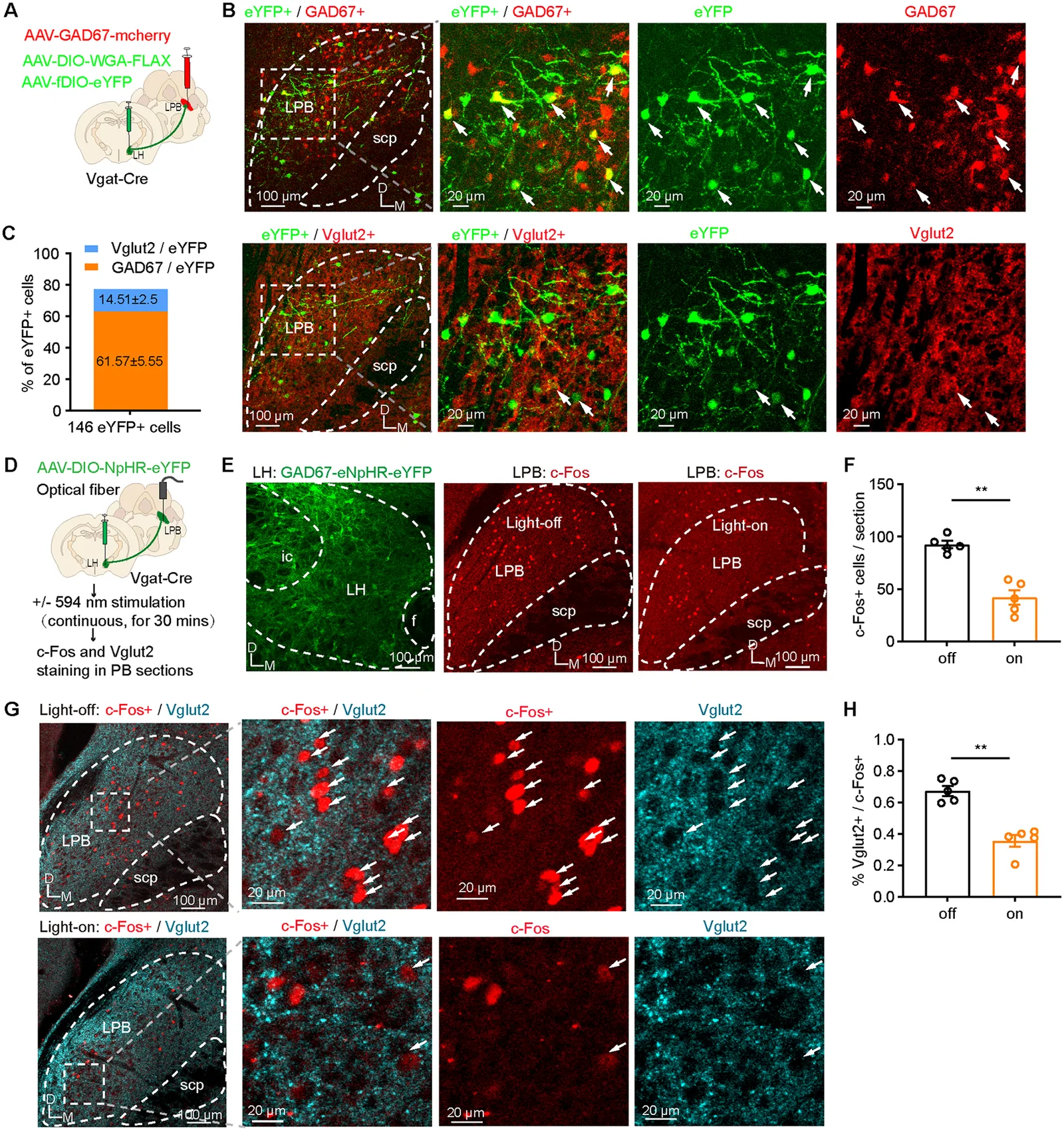
LH GABAergic projections primarily target LPB GABAergic neurons and modulate LPB glutamatergic activity via disinhibition. **(A)** Schematic diagram illustrating the experimental strategy for characterizing the output connectivity of LH GABAergic neurons in the LPB. **(B)** Representative images showing the co-localization of eYFP (labeling LH-innervated LPB neurons, green) with either GAD67 (GABAergic marker, red, upper panel) or Vglut2 (glutamatergic marker, red, lower panel). White arrows indicate representative double-labeled neurons (eYFP+/GAD67+ or eYFP+/Vglut2+). **(C)** Bar graph quantifying the percentage of eYFP-neurons in the LPB that are GAD67-positive (GABAergic) or Vglut2-positive (glutamatergic). Of the total identified eYFP-neurons (Total: 146 cells, *n* = 5 mice), a predominant proportion (~61.57%) were GAD67-positive (GABAergic neurons), while a smaller proportion (~14.51%) were Vglut2-positive (glutamatergic neurons). **(D)** Schematic diagram illustrating the experimental strategy in SNI Vgat-Cre mice. AAV-EF1α-DIO-NpHR-eYFP was injected into the LH, an optical fiber was implanted above the LPB for optogenetic inhibition of the LH-LPB GABAergic projection. **(E, F)** Representative images (E) and quantification (F) of c-Fos expression in the LPB under light-off and light-on conditions (F, *t* = 6.58, *P* = 0.0002, *n* = 5 mice). **(G, H)** Representative images (G) and summary data (H) showing the co-localization of c-Fos (red) and Vglut2 (cyan) in the LPB, where white arrows indicate double-labeled neurons (c-Fos + /Vglut2+) (H, *t* = 6.44, *P* = 0.0002; *n* = 5 mice). Data are presented as mean ± SEM. ** *P* < 0.01, two-tailed unpaired *t t*est. Orientation arrows: D, dorsal; M, medial. Data are available in S1 Data as a part of Supporting information.

Following these procedures, we quantified the co-localization of eYFP (LPB neurons innervated by LH GABAergic neurons) with either GAD67-mCherry or Vglut2 (Fig 6B). Our analysis revealed that a majority of eYFP-positive neurons in the LPB (61.57%) were GAD67-mCherry positive. In contrast, a smaller proportion (14.51%) of eYFP-positive cells co-expressed Vglut2 (Fig 6B and 6C). These findings indicate that LH GABAergic projections predominantly innervate inhibitory interneurons within the LPB, suggesting a potential disinhibitory mechanism for modulating LPB output.

Building upon the above anatomical findings, we next investigated the functional impact of these projections on LPB neuronal activity. First, we verified the activation status of the LH-LPB GABAergic projection in the neuropathic pain state and observed that SNI mice exhibited a significantly higher proportion of c-Fos-positive projection neurons compared to sham controls (S5A–S5D Fig), confirming that this specific pathway is functionally recruited during chronic pain. We then performed optogenetic inhibition of this pathway in SNI mice by injecting AAV-EF1α-DIO-NpHR-eYFP into the LH of SNI Vgat-Cre mice and implanted an optical fiber above the LPB, allowing for specific light-mediated inhibition of LH GABAergic terminals within this region (Fig 6D). In the light-on group, SNI mice received 30 min continuous yellow light (589 nm) stimulation, while the light-off control group received no light. Both groups were then subjected to c-Fos- and Vglut2-immunostaining in LPB sections (Fig 6D, 6E). Quantification revealed a significant reduction in the number of c-Fos-positive cells in the LPB in the optogenetically inhibited mice compared to the control mice (Fig 6F). More importantly, the percentage of LPB neurons co-expressing c-Fos and Vglut2 was significantly decreased in mice subjected to yellow light illumination of the LPB, relative to those subjected to no light (Fig 6G and 6H).

Together, these anatomical and functional data demonstrate that the LH-LPB GABAergic pathway, which is recruited during neuropathic pain, promotes the activation of excitatory LPB neurons likely through disinhibition of GABAergic neurons.

### LH GABAergic neurons modulate LPB glutamatergic neurons via a disinhibitory mechanism and subsequent nociception

To further elucidate the functional connectivity between LH GABAergic neurons and LPB neurons and investigate their role in nociceptive processing, we employed an optogenetic activation and fiber photometry recording strategy.

We first investigated the effect of LH GABAergic neuron activation on LPB glutamatergic neurons, we selectively expressed the excitatory opsin ChrimsonR-mCherry in LH GABAergic neurons (AAV-hSyn-DIO-ChrimsonR-mcherry into the LH) and GCaMP6s in LPB glutamatergic neurons (AAV-CaMKII-GCaMP6s into the LPB) in Vgat-Cre mice. An optical fiber, implanted above the LPB, enabled simultaneous optogenetic stimulation (633 nm) of ChrimsonR-expressing LH GABAergic terminals within the LPB and fluorescence recording (470 nm excitation) of GCaMP6 signals from LPB glutamatergic neurons (Fig 7A–7C). Successful viral expression and fiber implantation were confirmed by representative fluorescence images (Figs 7B; S6A, and S6B).

**Fig 7:**
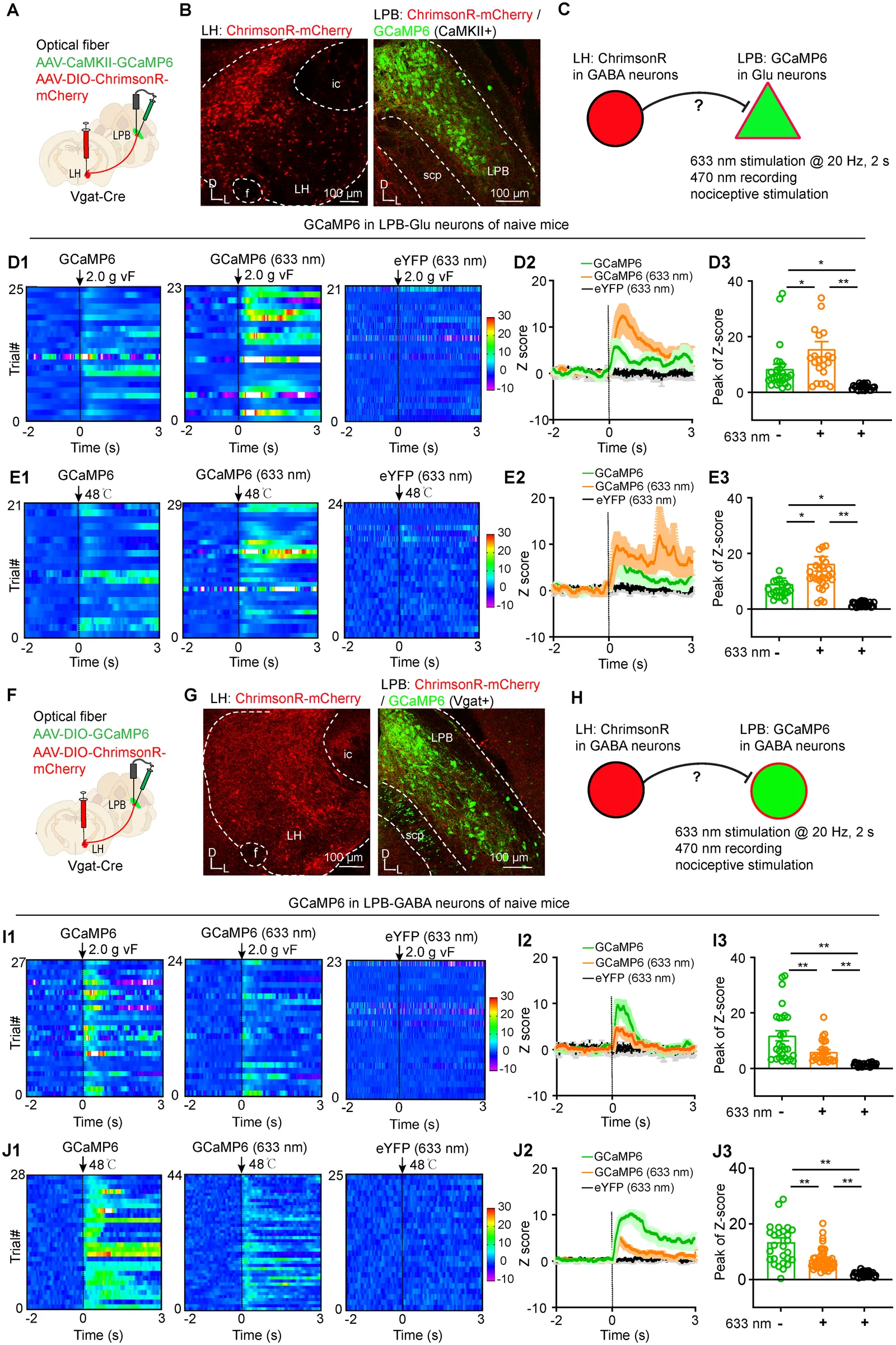
Optogenetic activation of LH GABAergic neurons differentially regulates responses in LPB glutamatergic and GABAergic neurons to nociceptive stimuli. **(A)** Schematic diagram of the experimental setup for optogenetic activation of the LH-LPB GABAergic projection (633 nm) and calcium recordings (470 nm) in the LPB. **(B)** Representative fluorescence images showing ChrimsonR-mCherry expression in LH GABAergic neurons (left) and GCaMP6 expression in LPB glutamatergic neurons (right). **(C)** Experimental paradigm for examining the projection from LH GABAergic neurons to LPB glutamatergic neurons. **(D1–D3)** Heatmaps (D1), average z-score (D2) and peak z-score (D3) of responses in GCaMP6 and eYFP signals to 2.0 g von Frey (vF) stimulation with (orange) and without (green) optogenetic stimulation with 633 nm laser (D3: *F*_(2, 66)_ = 12.27, *P* < 0.0001; *n* = 21-25 trials per group). **(E1–E3)** Heatmaps (E1), average z-score (E2) and peak z-score (E3) of responses in GCaMP6 and eYFP signals to 48 °C thermal stimulation with (orange) and without optogenetic stimulation (green) with 633 nm laser (E3: *F*_(2, 71)_ = 13.88, *P* < 0.0001; *n* = 21-29 trials per group). **(F)** Schematic of experimental setup for optogenetic activation of the LH-LPB GABAergic projection (633 nm) and calcium recordings (470 nm) in LPB GABAergic neurons. **(G)** Representative fluorescence images showing ChrimsonR-mCherry expression in LH GABAergic neurons (left) and GCaMP6 expression in LPB GABAergic neurons (right). **(H)** Experimental paradigm for examining the projection from LH GABAergic neurons to LPB GABAergic neurons. **(I1–I3)** Heatmaps (I1), average z-score (I2) and peak responses (I3) of GCaMP6 and eYFP signals to 2.0 g von Frey (vF) stimulation with (orange) and without optogenetic stimulation (green) with 633 nm laser (I3: *F*_(2, 71)_ = 16.66, *P* < 0.0001; *n* = 23-27 trials per group). **(J1–J3)** Heatmaps (J1), average Z-score (J2) and peak z-score (J3) of responses in GCaMP6 and eYFP signals to 48 °C thermal stimulation with (orange) and without optogenetic stimulation (green) with 633 nm laser (J3: *F*_(2, 94)_ = 30.64, *P* < 0.0001; *n* = 25-44 trials per group). Data are presented as mean ± SEM. * *P* < 0.05; ** *P* < 0.01. One-way ANOVAs with Tukey’s post-hoc tests were used in panels (D3, E3, I3, J3). The vertical red line indicates the presence of 633 nm stimulation. Orientation arrows: D, dorsal; L, lateral. Data are available in S1 Data as a part of Supporting information.

Both 2.0 g von Frey stimulation and 48 °C thermal stimulation evoked robust increases in GCaMP6s signal in LPB glutamatergic neurons (Fig 7D1, left; Fig 7E1, left). Optogenetic activation of LH GABAergic neuron terminals (633 nm light) significantly augmented these GCaMP6s responses to both mechanical and thermal stimuli, whereas eYFP controls showed no significant changes (Fig 7D1–7E3). These findings suggest that LH GABAergic input to LPB glutamatergic neurons enhances their excitability during noxious mechanical and thermal challenges.

Next, we investigated the effect of LH GABAergic neuron activation on LPB GABAergic neurons. This involved modifying our recording strategy to express AAV-EF1α-DIO-GCaMP6s specifically in LPB GABAergic neurons (in LPB of Vgat-Cre mice), while transfecting ChrimsonR-mCherry into LH GABAergic neurons (Figs 7F–7H, S6C, and S6D). In this setup, both 2.0 g von Frey stimulation and 48 °C thermal stimulation elicited robust GCaMP6s responses in LPB GABAergic neurons (Fig 7I1, left; Fig 7J1, left). However, optogenetic activation of LH GABAergic terminals significantly reduced GCaMP6s responses in LPB GABAergic neurons to both von Frey and thermal stimulation (Fig 7I1–7J3). This provides evidence for a direct inhibitory input from LH GABAergic neurons onto LPB GABAergic interneurons.

Consistent with these findings, in the absence of external sensory stimuli, optogenetic stimulation of LH GABAergic terminals in the LPB directly evoked a decrease in calcium transients in LPB GABAergic neurons, while simultaneously inducing an increase in the activity of LPB glutamatergic neurons (S6E–S6G Fig). Moreover, in SNI mice, optogenetic activation of LH GABAergic terminals similarly potentiated responses in LPB glutamatergic neurons while attenuated responses in LPB GABAergic neurons to both von Frey and thermal stimulation (S7A1–S7D3 Fig).

These results suggest that LH GABAergic input modulates nociceptive processing in the LPB primarily by targeting local GABAergic interneurons. By reducing the activity of these inhibitory interneurons, the LH GABAergic inputs effectively boost LPB glutamatergic neurons’ responses to nociceptive stimuli.

### LH-LPB GABAergic projections mediate pain modulation via a disinhibitory mechanism

To investigate the role of LH-LPB GABAergic projections and their interaction with LPB GABAergic neurons in pain modulation, we employed an approach combined optogenetic with chemogenetic technique. Specifically, AAV-EF1α-DIO-ChR2-eYFP was injected into the LH of Vgat-Cre mice to express ChR2 in GABAergic neurons and optical fiber was implanted above the LH, allowing for optogenetic activation of their projections in the LPB (S8A and S8B Fig). Simultaneously, AAV-EF1α-DIO-hM3Dq-mCherry (or AAV-EF1α-DIO-mCherry as a control) was injected into the LPB to enable chemogenetic activation of local GABAergic neurons via intraperitoneal administration of clozapine-N-oxide (CNO, 1 mg/kg) (Fig 8A). Immunofluorescence confirmed successful viral expression and the anatomical overlap within the LPB, showing ChR2-eYFP-labeled axonal fibers and hM3Dq-mCherry-labeled LPB GABAergic cell bodies (Fig 8B).

**Fig 8:**
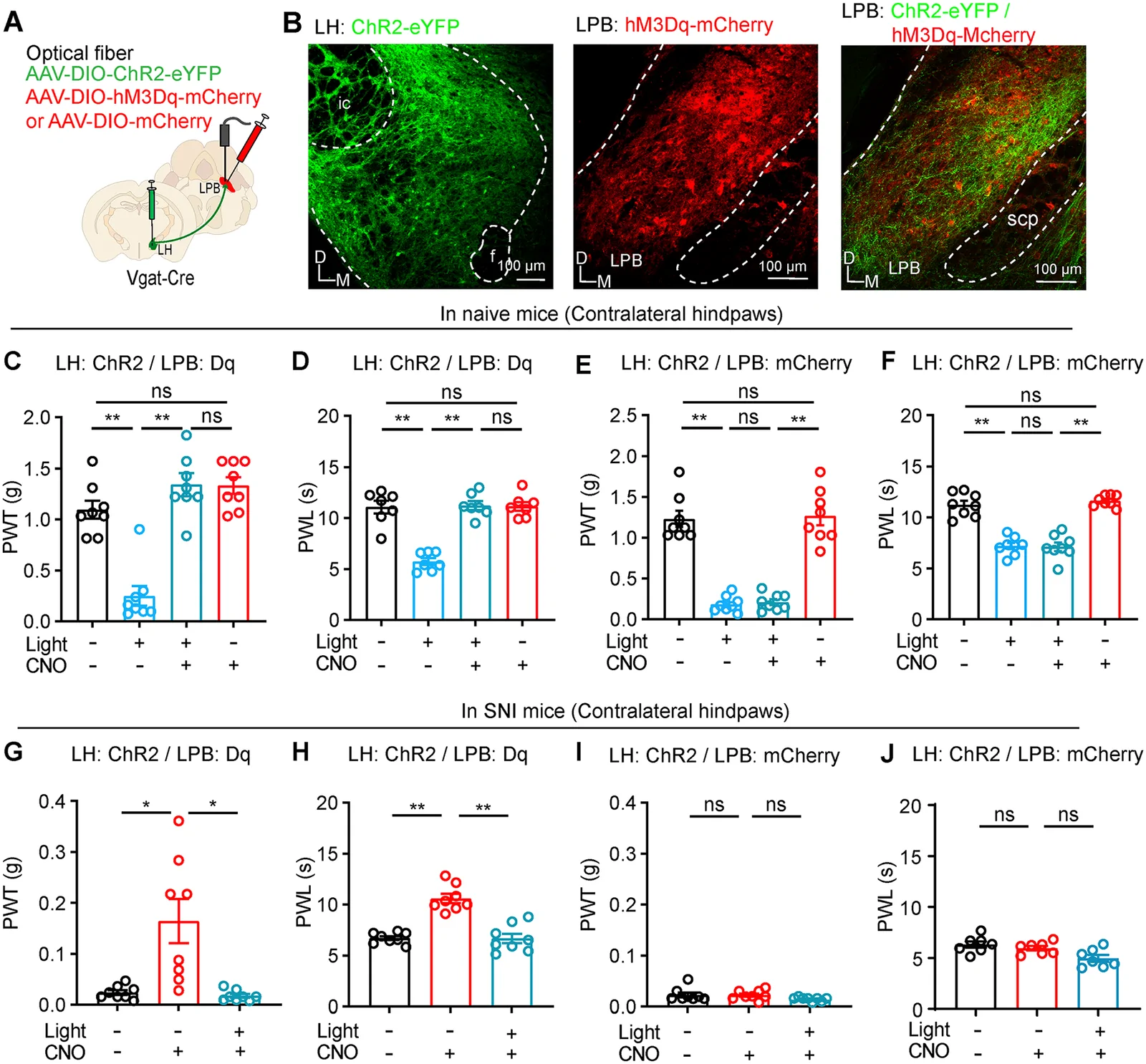
Optogenetic excitation of the LH-LPB GABAergic projection and its interaction with LPB GABAergic neurons in pain regulation. **(A)** Schematic diagram illustrating the experimental strategy. ChR2 was virally transfected into GABAergic neurons of the LH and an optical fiber was implanted into the LPB. Excitatory DREADD, hM3Dq, was virally transfected into LPB GABAergic neurons, which can be activated by intraperitoneal administration of 1 mg/kg CNO. **(B)** Representative images showing the expression of ChR2-eYFP in LH GABAergic neurons (left), hM3Dq-mCherry in LPB neurons (middle), and the distribution of LH-originating eYFP+ axonal terminals among mCherry+ neurons in the LPB (right). **(C–F)** Behavioral testing in naïve mice (contralateral hind paws). **(C, D)** Mechanical PWT (C) and thermal PWL (D) in the ChR2/Dq group under different combinations of optogenetic (Light) and chemogenetic (CNO) stimulation (C, *F*_(2.05, 14.34)_ = 32.65, *P* < 0.0001; *n* = 8 per group. D, *F*_(2.28, 13.56)_ = 63.41, *P* < 0.0001; *n* = 7 per group). **(E, F)** Mechanical PWT (E) and thermal PWL (F) in the ChR2/mCherry control group under identical stimulation conditions (E, *F*_(2.29, 16.00)_ = 66.73, *P* < 0.0001; *n* = 8 per group. F, *F*_(2.54, 17.76)_ = 58.77, *P* *<* 0.0001; *n* = 8 each group). **(G–J)** Behavioral testing in SNI mice (contralateral hind paws). **(G, H)** Mechanical PWT (G) and thermal PWL (H) in the ChR2/Dq group post-SNI surgery, showing the interaction between LH projection activation and LPB GABA neuron activation (G, *F*_(1.04, 7.25)_ = 11.55, *P =* 0.01; *n* = 8 per group. H, *F*_(1.80, 12.61)_ = 47.34, *P* < 0.0001; *n* = 8 per group). **(I, J)** Mechanical PWT (I) and thermal PWL (J) in the ChR2/mCherry control group under the same SNI conditions (I, *F*_(1.92, 13.40)_ = 2.99, *P* = 0.086; *n* = 8 per group. J, *F*_(1.83, 10.98)_ = 9.37, *P* = 0.0048; *n* = 7 per group). Data are presented as mean ± SEM. * *P* < 0.05, ** *P* < 0.01. One-way ANOVAs with Tukey’s post-hoc tests were used for panels **(C–J)**. Orientation arrows: D, dorsal; M, medial. Data are available in S1 Data as a part of Supporting information.

In naive mice, testing the hind paws contralateral to the virus injection side (Fig 8C–8F), optogenetic activation of LH-LPB GABAergic projections significantly reduced mechanical PWT and thermal PWL, indicating a pro-nociceptive effect (Fig 8C and 8D, Light+/CNO−). While chemogenetic activation of LPB GABAergic neurons with CNO alone did not alter baseline thresholds, it significantly attenuated the pain-like effects induced by LH-LPB projection stimulation (Fig 8C and 8D, Light+/CNO+). In the control group where mCherry was expressed in the LPB, CNO administration failed to block the pro-nociceptive effects of light stimulation; importantly, light stimulation persistently induced significant hypersensitivity in these mice regardless of CNO treatment (Fig 8E and 8F).

We then extended this investigation to SNI mice (Fig 8G–8J). In the hM3Dq-expressing group, chemogenetic activation of LPB GABAergic neurons with CNO alone significantly increased both mechanical PWT and thermal PWL in the contralateral injured paws, indicating a significant analgesic effect (Fig 8G and 8H, Light−/CNO+). Critically, these CNO-induced anti-nociceptive effects were significantly antagonized by the simultaneous optogenetic activation of LH GABAergic projections (Fig 8G and 8H, Light+/CNO+). In SNI mCherry mice, neither CNO alone nor CNO + light had any effect on mechanical or thermal hypersensitivity (Fig 8I and 8J).

Together, these behavioral results demonstrate that the effects of LH GABAergic projections are critically mediated by their interaction with LPB GABAergic neurons. In naive mice, LH GABAergic activation induces pain hypersensitivity, which is blocked by LPB GABAergic neuron activation. Conversely, in the SNI model, the analgesia produced by LPB GABAergic activation is antagonized by LH GABAergic input activation. This highlights a complex, context-dependent modulatory role of the LH-LPB GABAergic circuit, supporting a disinhibitory mechanism in pain processing.

## Discussion

In this study, we systematically dissected the distinct anatomical and functional roles of parallel projections from LH GABAergic neurons to the LPB and PVT in modulating pain and affect. Our findings reveal a distinct functional segregation: LH-PVT GABAergic projections bidirectionally modulate anxiety-like behaviors and affective states, with their inhibition exerting notable analgesic and anxiolytic effects in neuropathic pain. In contrast, LH-LPB GABAergic projections selectively and unilaterally regulate nociception across both physiological and neuropathic pain conditions, primarily through a disinhibitory mechanism involving local LPB GABAergic interneurons that ultimately potentiates pain responses in LPB glutamatergic neurons. These findings offer critical insights into the hypothalamic regulation of pain perception and its affective dimensions, highlighting a functionally segregated control over distinct aspects of pain.

Previous studies have implicated the LH GABAergic output in the intricate processing of reward and aversion [18–21]. Our investigation extends this by revealing that activation of the LH-PVT GABAergic projection promoted anxiety and aversion, suggesting that a unique role for these outputs to the PVT in maintaining a pro-anxiety state. Critically, the inhibition of this projection yielded significant analgesic and anxiolytic effects on neuropathic pain, highlighting the LH-PVT GABAergic pathway as an important circuit contributing to pain-affect comorbidity. Our anatomical evidence further supports this broad influence, as the LH GABAergic fibers are uniformly distributed along the anteroposterior axis of the PVT, ensuring a consistent modulation across its different subregions.

The observation that inhibiting the LH-PVT GABAergic projection produces analgesic and anxiolytic effects is contrast to the reported function of the PVT. Literature reports that activation of PVT glutamatergic neurons and their outputs to the vmPFC or BLA is associated with increased anxiety and negative affect [12,23,24]. This contradiction likely reflects the unique electrophysiological properties of PVT neurons. Sustained GABAergic inhibition hyperpolarizes and silences these neurons, meanwhile de-inactivates T-type calcium channels, which promotes rebound burst firing [25]. The latter may potently drive downstream anxiety-related nuclei. In fact, the PVT is a diverse nucleus where distinct neuronal populations exert opposite effects on emotional processing [12,26]. For instance, activation of PVT neurons receiving oxytocinergic inputs from the PVN or projections from the preBötC relieve anxiety [27,28]. In this study, we modulated PVT neurons inhibited by LH GABAergic neurons, probably resulting in different outcomes from modulation of PVT neurons in general. Such precise modulation likely influences specific subpopulations or alters the excitatory/inhibitory balance within distinct circuit motifs [29]. Collectively, these results demonstrate that the LH-PVT pathway recruits complex mechanisms—potentially involving firing-mode switching or disinhibition—that significantly contribute to the growing understanding of the PVT’s functional complexity in pain and emotional processing.

The fiber photometry data showing robust activation of LH-PVT GABAergic terminals by noxious, aversive, and even rewarding stimuli further supports its broad involvement in processing salient environmental cues. These results suggest that LH-PVT GABAergic terminals encode overall salience, and their dysregulation may lead to heightened negative affect and pain. The finding that the analgesic and anxiolytic effects via inhibiting this pathway were observed in neuropathic pain, but not in naive animals, underscores its specific involvement in chronic pain conditions. This suggests that LH-PVT GABAergic pathway may be a promising target for therapeutic interventions for alleviating both the sensory and affective dimensions of neuropathic pain.

In stark contrast to the LH-PVT GABAergic pathway, this study revealed that the LH-LPB GABAergic projection primarily and unilaterally regulates nociception across both physiological and neuropathic pain states, with minimal influence on general affective or rewarding/aversive behaviors. While the LPB is a well-established core processing nociceptive, aversive, and emotional information [9,30–32], our calcium imaging data showed multi-modal responses in LH-LPB GABAergic terminals, while modulation of these terminals only altered pain thresholds, but not emotional states. Recent studies have demonstrated that distinct LPB neuronal populations, defined by their genetic markers and projection targets, separately encode the sensory and affective components of pain [4,5,8,33]. The LH-LPB GABAergic input may selectively modulate the sub-circuits dedicated to nocifensive signaling, while the broader affective-aversive states may require activation of LPB neurons projecting to nuclei such as the amygdala or BNST [8]. Our findings thus highlight that under the specific input of the LH, the LPB’s GABAergic circuitry may be selectively harnessed for the precise modulation of pain sensation, rather than engaging emotion-related functions.

It is worth noting that a small fraction of LH GABAergic neurons (~7.5%) send collateral projections to both the PVT and LPB. These dual-projecting neurons may provide a structural basis for the co-regulation of pain and emotion. By simultaneously engaging both targets, this subpopulation could synchronize physiological nociception with negative affective states, ensuring a unified response to noxious stimuli.

Our anatomical and functional characterization of the LH-LPB circuitry uncovered a novel mechanism: LH GABAergic neurons predominantly project to and inhibit local GABAergic interneurons within the LPB. This disinhibitory action, by reducing local inhibition, effectively potentiates the activity of LPB glutamatergic projection neurons, thereby enhancing nociceptive signaling. Notably, this innervation is not uniform but follows a distinct anatomical gradient, with a significantly higher density of LH GABAergic fibers targeting the anterior subregions of the LPB. This intricate synaptic arrangement, coupled with such subregional targeting, provides a sophisticated means by which the LH can fine-tune pain transmission. A crucial aspect of this pathway’s function is its pronounced laterality. Our behavioral experiments demonstrated that acute activation of the LH-LPB pathway induces pronociception (hyperalgesia and allodynia) and its inhibition produces analgesia—both effects showing strong laterality, primarily affecting the contralateral side of the body. This lateralized control of pain transmission aligns with previous findings from our group and others, where distinct brain circuits, such as the projection from the subthalamic nucleus to the LPB, have been shown to modulate contralateral pain perception in chronic pain conditions [34]. This consistent lateralization across different forebrain-LPB pathways highlights a fundamental organizational principle for nociceptive processing in the brain.

Furthermore, the involvement of local GABAergic interneurons in shaping LPB output has been increasingly recognized in pain and aversion processing, with the balance between GABAergic inhibition and glutamatergic excitation determining nociceptive signal strength and valence [6,35]. Our findings reveal a specific top-down mechanism through which the LH modulates this local balance: LH GABAergic neurons predominantly inhibit local LPB GABAergic interneurons, thus disinhibiting and ultimately enhancing the activity of LPB glutamatergic projection neurons to contribute to nociceptive signaling. Importantly, while chemogenetic activation of LPB GABAergic neurons did not affect basal nociceptive thresholds, it effectively blocked neuropathic pain-like hypersensitivity, consistent with previous reports [6,8]. These converging lines of evidence underscore the critical role of LPB internal circuitry, particularly its GABAergic components, in fine-tuning pain transmission and pain relief.

The assessment of LH-LPB modulatory efficacy via fiber photometry should consider the profound hypersensitivity in SNI mice. We employed suprathreshold mechanical stimuli for naïve (2 g) and SNI (0.4 g) mice. Under these conditions, the LH-LPB projection maintained significant regulatory control over nociceptive signaling. While the baseline hyperactivity of LH GABAergic neurons in SNI mice confirmed by our c-Fos-staining data suggests this circuit enters an activated state during chronic pain, its modulatory impact on LPB calcium activity remains stable.

While our study provides significant insights, several limitations warrant consideration. Firstly, although we employed Vgat-Cre mice to selectively target GABAergic neurons, the LH itself is highly heterogeneous, containing various subtypes of GABAergic neurons with potentially distinct input-output profiles that were not fully resolved [36–38]. Secondly, while we characterized the functional impact of LH GABAergic inputs on PVT and LPB neurons, the downstream targets of these modulated PVT and LPB neurons, which ultimately drive the observed behavioral changes, were not directly elucidated. Thirdly, most of our behavioral manipulations involved acute optogenetic stimulation or inhibition; future investigations should explore the long-term effects of modulating these pathways on chronic pain and affect.

In conclusion, our study elucidates the distinct roles of two parallel LH GABAergic projections: the LH-PVT and LH-LPB pathways. We demonstrate that the LH-PVT pathway bidirectionally modulates anxiety and affective states, providing analgesic and anxiolytic effects upon inhibition in neuropathic pain (S9 Fig). Concurrently, the LH-LPB pathway precisely and unilaterally regulates nociception via a novel disinhibitory mechanism within the LPB (S9 Fig). These findings reveal the hypothalamus’s sophisticated role in differentially modulating the sensory and affective dimensions of pain, identifying these specific circuits as promising, pathway-specific targets for novel therapeutic strategies against chronic pain and its comorbidities.

## Materials and methods

### Ethics statement

All procedures for animal care and experimentation (Protocol No. 202207S123) were reviewed and approved by the Institutional Animal Care and Use Committee and the Office of Laboratory Animal Resources at Xuzhou Medical University, in accordance with the Regulations for the Administration of Affairs Concerning Experimental Animals (1988, China).

### Animals

Male and female Vgat-Cre (Jackson Laboratory, stock No. 017535) and wild-type C57BL/6 mice were used in approximately equal numbers and maintained in the university’s animal facility in a controlled environment (12 h light/dark cycle, 21–22 °C, 40%–70% humidity) with free access to food and water. All surgeries were performed on mice aged 8 weeks or older.

### Viral constructs

A series of adeno-associated viral (AAV) vectors were obtained from Brain VTA (Wuhan, China), including but not limited to: AAV-EF1α-DIO-hChR2(H134R)-eYFP, AAV-EF1α-DIO-NpHR-eYFP, AAV-EF1α-DIO-eYFP, AAV-EF1α-DIO-GCaMP6s, AAV-CaMKII-ChR2-eYFP, AAV retro-hSyn-mCherry, AAV retro-hSyn-eGFP, AAV-hSyn-DIO-WGA-Flp, AAV-hSyn-fDIO-eYFP, AAV-EF1α-DIO-mCherry, AAV-GAD67-mCherry, AAV-EF1α-DIO-hM4Di-mCherry, and AAV-EF1α-DIO-hM3Dq-mCherry. Titers for AAVs were 2 × 10^12^ to 5 × 10^12^ vg/ml.

### Stereotaxic surgery

All stereotaxic procedures for viral vector injection and optical fiber implantation were conducted under isoflurane anesthesia (3% induction, 1.5% maintenance) on a stereotaxic frame (RWD Life Science, Shenzhen, China), with mice maintained at constant body temperature on a heating pad [39,40]. Following skull exposure and disinfection, viral vectors were unilaterally injected (50 nL/min via 10 µL Hamilton syringe, KD Scientific, Holliston, MA, USA) into target brain regions using the following coordinates (relative to bregma, in mm): LH: AP: −0.94; ML: ±1.15; DV: −5.10; PVT: AP: −1.40; ML: ±1.15; DV: −3.02 (6° angle); LPB: AP: −5.15; ML: ±1.32; DV: −3.50. For optogenetic or fiber photometry experiments, optical fibers (200 µm diameter, NA 0.37, Inper, Hangzhou, China) were implanted 0.2 mm dorsal to the injection site. Post-operative analgesia was provided by administering meloxicam (4 mg/kg, Aladdin, Shanghai, China) in drinking water for three days.

Following the completion of behavioral experiments, brain sections (30–40 µm) were prepared to verify viral expression and optical fiber placement. Viral expression of ChR2, NpHR, ChrimsonR, GCaMP6, or hM3Dq in the target regions (LH, PVT, or LPB) was confirmed via fluorescence microscopy, as detailed in the corresponding Results sections and Figure panels. The specific localization of optical fiber tips was identified by brightfield imaging of the fiber tracks, and the integrated tip positions for all experimental animals were mapped onto the brain atlas templates to ensure anatomical consistency (S3K–S3M, S4A–S4G, S6A–S6D, S8A, and S8B Fig). Only data from mice with robust viral expression restricted to the intended nuclei and precise fiber tip placement within the anatomical boundaries of the PVT or LPB were included in the final statistical analysis.

### Spared nerve injury of the sciatic nerve

Neuropathic pain mouse model was established with spared nerve injury (SNI) of the sciatic nerve following a standard protocol [34,41]. Under isoflurane anesthesia, the sciatic nerve was exposed and two branches (the common peroneal and tibial nerves) were ligated and transected, leaving the third branch (the sural nerve) intact. Sham controls underwent nerve exposure without damage. Mice recovered on a heating pad after surgery until woke up, then were placed in a clean cage.

### von Frey filament test

Mechanical sensitivity was assessed using von Frey filaments (0.01–2 g) after habituation on a mesh platform at least for 1 h. Filaments were applied to the plantar of the hind paw, and withdrawal thresholds were determined using the up-down method [34,39,41].

### Hargreaves’ test

After 1 h habituation on a glass surface, thermal sensitivity was measured using the Hargreaves’ apparatus (Boerni, Tianjin, China). Radiant heat was applied to the hind paw and withdrawal latencies were recorded over three trials per paw, and the average was used for analysis [41,42].

### The open field test

The open field test was conducted in a standard arena (typically 40 cm × 40 cm × 40 cm). Each mouse was gently placed in the center and allowed a 6-minute free exploration period. Locomotor activity was meticulously tracked using the EthoVision XT system (Noldus Information Technology, Wageningen, the Netherlands). Key parameters, including total distance traveled, entries into, and time spent within the central zone, were subsequently quantified to evaluate both general activity and anxiety-like behaviors. Between successive trials, the arena was thoroughly cleaned with 75% ethanol to eliminate olfactory cues.

### Elevated plus maze (EPM)

The EPM assay was utilized to assess anxiety-like behaviors. The apparatus comprised two opposing open arms and two opposing closed arms (each 30 cm long, 5 cm wide), elevated 50 cm above the floor. Mice were individually placed at the central junction, facing an open arm, and permitted free exploration for 6 min. Anxiety-like behavior was quantified by measuring the number of entries into and total time spent in the open arms. All behavioral data were automatically recorded and analyzed using EthoVision XT software (Noldus Information Technology, Wageningen, the Netherlands). The maze was thoroughly cleaned with 70% ethanol between each trial to eliminate olfactory cues.

### Conditioned place preference (CPP) test

CPP or aversion was evaluated using a two-chamber apparatus with distinct visual and tactile cues. Initially, mice underwent a 20-minute pre-conditioning session to assess baseline chamber preference; those with >75% initial preference for either chamber were excluded. To avoid ceiling or floor effects, a counterbalanced preference-based assignment was used: for ChR2-mediated activation (expected to be aversive), light was paired with the initially preferred side; for NpHR-mediated inhibition (expected to be rewarding/anti-nociceptive), light was paired with the initially non-preferred side.

Conditioning involved pairing one chamber with an optogenetic light stimulation (blue light: 20 Hz, 5 ms, 4 mW; yellow light: 3 mW constant) for 30 min, delivered in cycles of 120 s ON/ 60 s OFF. The other chamber received no stimulation. Twenty-four hours post-conditioning, a test session (free access to both chambers, no stimulation) was conducted. Time spent in the conditioned chamber was automatically recorded and analyzed with EthoVision XT. Chamber preference or aversion was determined by comparing time spent in stimulus-paired versus unpaired chambers before and after conditioning.

### Fiber photometry

Neuronal activity within the LH was monitored using a fiber photometry system (ThinkerTech, Nanjing, China). A 470 nm LED (typically < 0.05 mW at fiber tip) excited the GCaMP6 indicator, while an isosbestic 405 nm LED provided a control signal to account for photo-bleaching and motion artifacts. Raw fluorescence signals were digitized at 1,000 Hz. For subsequent analysis, raw traces were first down-sampled offline to 100 Hz. The 405 nm signal was then utilized to correct the GCaMP6 signal for non-neuronal fluctuations. To quantify activity changes, data were converted to z-scores using the formula: *Z* = (*F* − Mean_baseline_)/SD_baseline_, where Mean_baseline_ and SD_baseline_ were derived from a 3-second pre-event baseline period. The magnitude of the response was further quantified by calculating the peak of the z-score traces within a 5-second post-stimulus window.

### Brain slice electrophysiology

Brain slice electrophysiological recording was conducted with whole-cell patch-clamp recording [34,39,43]. Acute coronal slices (280 μm) containing the LH, PVT or LPB were prepared with a Leica VT-1200 vibratome (Nussloch, Germany) in ice-cold, oxygenated sucrose-based cutting solution (in mM) (85 NaCl, 75 sucrose, 2.5 KCl, 1.25 NaH_2_PO_4_, 4.0 MgCl_2_, 0.5 CaCl_2_, 24 NaHCO_3_, and 25 glucose). After 1 h incubation at 32 °C, slices were transferred to standard artificial cerebrospinal fluid (ACSF) at 26 °C for at least 30 min. ACSF contains (in mM) 125 NaCl, 2.5 KCl, 1.25 NaH_2_PO_4_, 1.2 MgCl_2_, 2.4 CaCl_2_, 26 NaHCO_3_, and 11 glucose.

Electrophysiological whole-cell patch-clamp recordings were performed utilizing a Nikon FN-1 upright microscope and a Molecular Devices system comprising a MultiClamp 700B amplifier, Digidata 1500B, and associated pClamp 10.7 software. Cells with a resting membrane potential above −40 mV or holding current greater than −50 pA were excluded. Optogenetic stimulation was achieved with 460 nm or 560 nm light (2 mW) delivered via a 200 µm (NA 0.37) optical fiber from Plexon. To investigate the pharmacological profile of blue-light-evoked IPSCs or confirm monosynaptic transmission, neurons were exposed to either 50 µM picrotoxin (PTX) or 0.3 µM TTX with 50 µM 4-AP, respectively.

### Immunohistochemistry and imaging

Mice were deeply anesthetized via CO_2_ inhalation before cardiac perfusion, first with PBS and then with 4% paraformaldehyde (PFA). Brains were carefully dissected, post-fixed in 4% PFA overnight at 4°C, and subsequently cryoprotected in 30% sucrose solution until they sank. Coronal sections (30 µm thick) were prepared using a Leica CM1950 cryostat (Nussloch, Germany).

For immunostaining, free-floating sections were initially blocked and permeabilized in a solution containing 10% donkey serum and 0.1% Triton X-100 for 1.5 hours at room temperature. Following this, sections were incubated with primary antibodies overnight at 4 °C. After extensive washes with PBS, Alexa Fluor-conjugated secondary antibodies were applied for 2 hours at room temperature. Sections were then thoroughly washed again with PBS and allowed to dry.

Immunostained brain slices were mounted onto glass slides, coverslipped with mounting medium (MeilunBio, Dalian, China, Cat No. MA0222), and imaged using an Olympus FV-1000 confocal microscope (Tokyo, Japan). All image processing and analysis were performed with ImageJ software (NIH, Bethesda, MD, USA).

Primary Antibodies: Rabbit anti-Vglut2 IgG (Proteintech, 19209-1-AP, 1:300); Guinea pig anti-c-Fos IgG (Synaptic Systems, 226004, 1:2,000); Rabbit anti-c-Fos IgG (Cell Signaling Technology, 2250S, 1:1,500); Rabbit anti-GABA IgG (Sigma-Aldrich, A2052, 1:500). Secondary Antibodies: Alexa 488/555/647 donkey anti-rabbit IgG (Jackson ImmunoResearch, 1:500); Alexa 488/555/647 donkey anti-guinea pig IgG (Jackson ImmunoResearch, 1:500).

### Statistical analysis and schematic illustrations

All analyses were performed with GraphPad Prism 8.0. Electrophysiological and fiber photometry data were processed with Clampfit 10.7 (Molecular Devices, San Jose, CA). Figures were composed using Adobe Illustrator 2020. Data are presented as mean ± SEM. Comparisons between two groups were made with paired or unpaired two-tailed *t*-tests. Multi-group comparisons with one-way or two-way ANOVAs (with repeated-measures as appropriate), followed by Tukey’s post-hoc test. Mann–Whitney *U*-test or nonparametric ANOVA was used when variance assumption was invalid. Details of statistical testing, sample size (*n*), and significance are provided in figure legends. Power analyses (StatMate 2.0) ensured group sizes were adequate for ≥80% statistical power.

Brain Schematic, icons (such as syringes), and experimental diagrams within the figures were manually rendered using Adobe Illustrator. The anatomical outlines and coordinates were defined with reference to the Allen Mouse Brain Common Coordinate Framework (CCFv3; 2017 Allen Institute for Brain Science) and The Mouse Brain in Stereotaxic Coordinates by Paxinos and Franklin (2nd edition).

## Supporting information

S1 Fig Anatomical distribution of LH GABAergic projections across the rostrocaudal axes of the PVT and LPB. **(A)** Schematic diagram showing the injection of AAV-DIO-ChR2-eYFP into the LH of Vgat-Cre mice. **(B)** Representative image showing the expression of ChR2-eYFP in LH GABAergic neurons. **(C)** Representative images of eYFP-labeled projection fibers in the anterior (AP: −0.34 mm) and posterior (AP: −1.58 mm) subregions of the PVT. **(D)** Quantification of fluorescence intensity of LH GABAergic fibers across the anteroposterior (AP) axis of the PVT (*t* = 2.03, *P* = 0.098, *n* = 6 mice, paired *t* test). **(E)** Representative images of eYFP-labeled projection fibers across the anterior (AP: −4.9 mm), middle (AP: −5.1 mm), and posterior (AP: −5.3 mm) levels of the LPB. (F) Quantification of fluorescence intensity of LH GABAergic fibers across the AP axis of the LPB (*F*_(1.61, 8.05)_ = 23.45, *P* = 0.0006, *n* = 6 mice, one-way repeated-measures ANOVA with Tukey post hoc tests). * *P* < 0.05, ** *P* < 0.01. ns, not significant. Data are available in S1 Data as a part of Supporting information.
(TIF)

S2 Fig Characterization of pain and anxiety-like behaviors in SNI mice. **(A, B)** Time course of mechanical paw withdrawal threshold (PWT) and thermal paw withdrawal latency (PWL) in mice subjected to SNI (red) and Sham (black) surgery. (A) PWT. F _(1, 42)_ = 694.7, *P* < 0.0001; *n* = 9 per group. (B) PWL. F _(1, 48)_ = 753.8, *P* < 0.0001; *n* = 9 per group). **(C, D)** Elevated plus maze (EPM) tests for SNI and Sham mice. (C) Frequency of entries into the open arms. *t* = 3.56, *P* = 0.0035; *n* = 7 in SNI, *n* = 8 in Sham. (D) Time spent in the open arms. *t* = 3.38, *P* = 0.0049; *n* = 7 in SNI, *n* = 8 in Sham. **(E–H)** Open field tests (OFT) for SNI and Sham mice. (E) Frequency of entries into the center area. *t* = 2.70, *P* = 0.016. (F) Duration in the center. *t* = 5.14, *P* = 0.0001. (G) Total distance traveled. *t* = 1.528, *P* = 0.15. (H) Velocity in the OFT. *t* = 1.53, *P* = 0.15. *n* = 9 in SNI, *n* = 8 in Sham. ** *P* < 0.01. ns, not significant. Two-way ANOVAs with post-hoc Bonferroni tests for panels (A, B). Two-tailed unpaired *t* test for panels (C–H). Data are available in S1 Data as a part of Supporting information.
(TIF)

S3 Fig Activation of the LH-PVT pathway induces anxiety-like behavior, while inhibition has no significant effect on pain or anxiety in naive mice. **(A1, A2)** Mechanical PWT in ChR2 (A1, F_(1, 18)_ = 1.96, *P* = 0.18, *n* = 10) and eYFP (A2, F_(1, 18)_ = 0.17, *P* = 0.69; *n* = 10) mice during light on (blue) and light off (black) for contralateral (Contra) and ipsilateral (Ipsi) hind paws. No significant differences were observed. **(B1, B2)** Thermal PWL in ChR2 (B1, *F*_(1, 14)_ = 0.0014, *P* = 0.97; *n* = 8) and eYFP (B2, F_(1, 14)_ = 0.20, *P* = 0.66; *n* = 8) mice during light on (blue) and light off (black) conditions for contralateral and ipsilateral hind paws. No significant differences were observed. **(C1)** Representative track plots in the OFT upon ChR2 activation from a ChR2 mouse and an eYFP mouse. **(C2, C3)** During blue light illumination, frequency (C2, *t* = 4.42, *P* = 0.0003) and duration (C3, *t* = 4.49, *P* = 0.0003) in the center of the OFT in ChR2 mice (*n* = 10) were significantly decreased, relative to eYFP mice (*n* = 10). **(C4, C5)** During blue light illumination, total distance traveled (C4, *t* = 0.22, *P* = 0.83) or velocity (C5, *t* = 0.22, *P* = 0.83) did not differ between ChR2 (*n* = 10) and eYFP (*n* = 10) mice. **(D1–D3)** Assessment of anxiety-like behavior upon blue light illumination in the EPM. (D1) Representative tracks in the EPM from a ChR2 mouse and an eYFP mouse. (D2–D3) Blue light illumination significantly decreased frequency (D2, *t* = 2.31, *P* = 0.034) and duration (D3, *t* = 3.56, *P* = 0.0024) in the open arms of the EPM in ChR2 mice (*n* = 9), relative to eYFP mice (*n* = 10). **(E1–E3)** CPP for ChR2 activation. (E1) Representative heatmaps showing moving tracks of a ChR2 mouse and an eYFP in pre-test and test conditions. Pairing of blue light illumination did not cause changes in time spent in the light side in ChR2 (E2, *t* = 1.60, *P* = 0.15, pre-test vs. test, *n* = 9) and eYFP mice (E3, *t* = 0.029, *P* = 0.98, pre-test vs. test, *n* = 8). **(F1, F2)** Mechanical PWT in NpHR (F1, F_(1, 18)_ = 2.009, *P* = 0.17; *n* = 10 per group) and eYFP (F2, F_(1, 18)_ = 0.42, *P* = 0.52; *n* = 10 per group) groups during light on (orange) and light off (black) conditions for contralateral and ipsilateral hind paws. **(G1, G2)** Thermal PWL in NpHR (G1, *F*_(1, 14)_ = 1.23, *P* = 0.29; *n* = 8 per group) and eYFP (G2, *F*_(1, 14)_ = 0.14, *P* = 0.71; *n* = 8 per group) mice during light on (orange) and light off (black) conditions for contralateral (Cintra) and ipsilateral (Ipsi) paws. **(H1–H5)** Assessment of anxiety-like behavior with open field tests (OFT). (H1) Representative tracks in the OFT from a NpHR mouse and an eYFP mouse during yellow light illumination. (H2-H3) No differences in frequency (H2, *t* = 0.028, *P* = 0.98) or duration (H3, *t* = 0.91, *P* = 0.37) in the center between NpHR (*n* = 9) and eYFP (*n* = 10) mice. (H4, H5) No significant differences in total distance traveled (H4, *t* = 1.93, *P* = 0.071) or velocity (H5, *t* = 1.93, *P* = 0.071) in the OFT during yellow light illumination in NpHR (*n* = 9) and eYFP (*n* = 10) mice. **(I1–I3)** Assessment of anxiety-like behavior in the EPM upon yellow light illumination. (I1) Representative tracks in the EPM from a NpHR mouse and an eYFP mouse. No significant differences in frequency (I2, *t* = 0.12, *P* = 0.92) or duration (I3, *t* = 1.40, *P* = 0.17) in the open arms of the EPM between NpHR (*n* = 9) and eYFP (*n* = 10) mice. **(J1–J3)** CPP in NpHR and eYFP mice. (J1) Representative moving track in CPP chamber in pretest and test sessions from a NpHR mouse and an eYFP mouse. (J2, J3) No significant differences in time spent in the light-paired side in NpHR mice (J2, *t* = 2.33, *P* = 0.053, pre-test vs. test, *n* = 8) and eYFP mice (J3, *t* = 0.32, *P* = 0.76, pre-test vs. test, *n* = 8). **(K)** Strategy for optogenetic modulation of LH-PVT pathway. **(L, M)** Representative images of fiber tracks and summarized fiber tip locations (circles) for ChR2 (L) and NpHR (M) cohorts. * *P* < 0.05; ** *P* < 0.01. Two-way ANOVA was used in panels (A1, A2, B1, B2, F1, F2, G1, G2) followed by post-hoc tests where appropriate. Unpaired Student’s *t*-tests were used in panels (C2–C5, D2, D3, H2–H5, I2, I3). Paired *t*-tests were used in panels (E2, E3, J2, J3) to compare pre-test vs. test within the same group. Data are available in S1 Data as a part of Supporting information.
(TIF)

S4 Fig Histological validation of viral expression and fiber placement. **(A, D, F)** Schematics of viral injection in the LH and optical fiber implantation in the LPB (A, F) or PVT (D) of Vgat-Cre mice. Left panels in **(B, C, E, G)**, representative images showing optical fiber tracks in the target regions. Right panels in (B, C, E, G): summarized fiber tip locations (circles) for all animals in each cohort.
(TIF)

S5 Fig Functional recruitment of LH-GABAergic projections to the LPB in SNI mice. **(A)** Representative image showing Retro-eYFP expression in the LPB. **(B)** Representative images of eYFP (green), c-Fos (cyan), and GABA (red) immunofluorescence in the LH of Sham (top) and SNI (bottom) mice. Arrows indicate triple-labeled neurons. **(C)** Percentage of eYFP+ neurons expressing both c-Fos and GABA (*t* = 2.74, *P* = 0.034, n = 4 mice per group). **(D)** Percentage of total eYFP+ neurons expressing c-Fos (*t* = 2.53, *P* = 0.045, *n* = 4 mice per group). Data are presented as mean ± SEM. * *P* < 0.05, unpaired *t* test. Data are available in S1 Data as a part of Supporting information.
(TIF)

S6 Fig Photometry verification of LH-GABAergic modulation on LPB neurons. **(A, C)** Schematic diagrams for fiber photometry recording of LPB neurons during optogenetic stimulation of LH-GABAergic terminals. **(B, D)** Representative brightfield images (left) and fiber tip positions (right) in the LPB for Vgat-GCaMP6 (B) and CaMKII-GCaMP6 (D) mice. **(E)** Representative heatmaps showing z-score changes of GCaMP6 or eYFP fluorescence in response to 633 nm laser stimulation. **(F)** Average calcium transients (z-score) of LPB neurons from Vgat-GCaMP6 (green), CaMKII-GCaMP6 (orange), and CaMKII-eYFP (black) groups. **(G)** Quantification of peak Z-scores following laser onset. F_(2, 57)_ = 110.6, *P* < 0.001, *n* = 21 in Vgat-GCaMP6, *n* = 17 in CaMKII-GCaMP6, *n* = 22 in eYFP. Data are presented as mean ± SEM. **P < 0.01, one-way ANOVA with Tukey’s post hoc test. Data are available in S1 Data as a part of Supporting information.
(TIF)

S7 Fig Optogenetic activation of LH GABAergic neurons modulates activity in LPB glutamatergic and GABAergic neurons in responses to nociceptive stimuli in SNI mice. **(A1–A3)** Heatmaps (A1), average z-score (A2) and peak z-scores (A3) of responses in GCaMP6 and eYFP signals of LPB-Glu neurons to 2.0 g von Frey stimulation on the hind paw in SNI mice with (orange) and without (green) optogenetic stimulation of the LH-LPB GABAergic projection with 633 nm laser. *F*_(2, 62)_ = 21.40, *P* < 0.0001; *n* = 27 trials in GCaMP6, *n* = 18 trials in GCaMP6 (633 nm), *n* = 20 trials in eYFP. **(B1–B3)** Heatmaps (B1), average z-score (B2) and peak z-scores (B3) of responses in GCaMP6 and eYFP signals in LPB-Glu neurons to 48 °C thermal stimulation on the hind paw of SNI mice with (orange) and without (green) optogenetic stimulation of the LH-LPB GABAergic projection with 633 nm laser. *F*_(2, 57)_ = 28.53, *P* < 0.0001; *n* = 21 trials in GCaMP6, *n* = 17 trials in GCaMP6 (633 nm), *n* = 22 trials in eYFP. **(C1–C3)** Heatmaps (C1), average z-score (C2) and peak z-scores (C3) of responses in GCaMP6 and eYFP signals in LPB-GABA neurons to 2.0 g von Frey stimulation on the hind paw of SNI mice with (orange) and without (green) optogenetic stimulation of the LH-LPB GABAergic projection with 633 nm laser. *F*_(2, 81)_ = 13.17, *P* < 0.0001; *n* = 30 trials in GCaMP6, *n* = 24 trials in GCaMP6 (633 mm), *n* = 30 trials in eYFP. **(D1–D3)** Heatmaps (D1), average z-score (D2) and peak z-scores (D3) of responses in GCaMP6 and eYFP signals in LPB-GABA neurons to 48 °C thermal stimulation on the hind paw of SNI mice with (orange) and without (green) optogenetic stimulation of the LH-LPB GABAergic projection with 633 nm laser. *F*_(2, 78)_ = 32.06, *P* < 0.0001; *n* = 32 trials in GCaMP6, *n* = 25 trials in GCaMP6 (633 mm), *n* = 24 trials in eYFP. *P < 0.05; **P < 0.01. One-way ANOVAs with Tukey’s post hoc tests were used in panels (A3, B3, C3, D3). Data are available in S1 Data as a part of Supporting information.
(TIF)

S8 Fig Histological validation of fiber placement in the LPB. **(A–B)** Schematics diagram of viral injection in the LH and optical fiber implantation in the LPB (A) of Vgat-Cre mice. (B) Representative micrograph of fiber track (left panel) and summarized fiber tip locations (circles) (right panel) in the LPB.
(TIF)

S9 Fig Schematic model of LH GABAergic circuits in pain and anxiety regulation. Diagram showing divergent LH GABAergic projections. Population 1 projects to the PVT, modulating both pain and anxiety. Population 2 projects to the LPB and modulates pain through a disinhibition mechanism (inhibiting local GABAergic neurons to activate glutamatergic neurons).
(TIF)

S1 Data Summary of underlying raw data. This Excel file contains the individual numerical values used to generate all graphs and statistical analyses presented in the manuscript. The data for each figure are organized into separate sheets.
(XLSX)

## Abbreviations

AAV: adeno-associated viral
ACSF: artificial cerebrospinal fluid
CPP: conditioned place preference
EPM: elevated plus maze
IPSCs: inhibitory postsynaptic currents
LH: lateral hypothalamus
OFT: open field test
PWL: paw withdrawal latency
PWT: paw withdrawal threshold
SNI: spared nerve injury
WT: wild-type
