# Supplementary material for: Two parallel lateral hypothalamic GABAergic projections differentially modulate pain and anxiety in mice
Source: PLoS Biol. 2026 Nov 5;24(11):e3004096. doi: 10.1371/journal.pbio.3004096 (PMC13739175; doi:10.1371/journal.pbio.3004096)
Supplement: Supplementary file 1 [file pbio.3004096.s011.docx]

**REVIEWER COMMENTS**

**Reviewer #1:**

*This study investigates two parallel GABAergic projection pathways from the LH to the PVT and the LPB and their differential modulation of pain and anxiety. The research reveals two functionally segregated neural circuits, providing new insights into the mechanistic basis of pain-emotion comorbidity, demonstrating significant innovation. However, the manuscript could be further improved in terms of image quality and result justification. We encourage additional revisions to strengthen the overall quality and academic rigor of the study. Specific comments are provided below.*
*1. Previous studies have demonstrated that the regulation of pain and emotion by the PVT and LPB exhibits subregion-specific differences. Therefore, do the projections and functions of the LH to the PVT and LPB also exhibit subregional specificity? It is recommended to supplement this with relevant experiments for clarification*

**Response:** We thank the reviewer for this insightful comment. We agree with the reviewer that subregional specificity is a key aspect of PVT and LPB function. To address this, we analyzed the distribution of LH-GABAergic fibers across the anteroposterior (AP) axis of both regions. Our anatomical data (S1 Fig) show that LH-PVT GABAergic projection is relatively uniform across the AP axis (P > 0.05); the LH-LPB GABAergic projection exhibits subregional differences, with higher innervation density in the anterior part of the LPB compared to its posterior part (P < 0.05). While the primary focus of this study remains the overall functional impact of these inhibitory inputs on pain-affect behaviors, these anatomical findings provide a crucial structural basis for understanding how the LH modulates these targets. We have now incorporated these distribution data into the Results (Lines 164-171) and discussed their potential functional implications in the Discussion (Lines 693-696, 750-754). The representative mapping and quantification are included in the revised Supplementary Information (S1 Fig).

In the Results section, we wrote: “Furthermore, we mapped the distribution of LH GABAergic fibers along the AP axis of the PVT and LPB by expressing ChR2-eYFP in LH GABAergic neurons in Vgat-cre mice (S1A–C and S1E Fig). Fluorescence intensity analysis revealed that the projection density was relatively consistent across the anterior and posterior PVT (S1D Fig). In contrast, the density of LH GABAergic fibers in the LPB showed a significant AP gradient with higher density in the anterior subregions compared to the posterior subregions (S1F Fig). These results indicate that the LH-LPB projection, but not the LH-PVT projection, exhibits distinct subregional patterns.”.

In the Discussion section, we wrote: “Our anatomical evidence further supports this broad influence, as the LH GABAergic fibers are uniformly distributed along the anteroposterior axis of the PVT, ensuring a consistent modulation across its different subregions.”.

And “Notably, this innervation is not uniform but follows a distinct anatomical gradient, with a significantly higher density of LH GABAergic fibers targeting the anterior subregions of the LPB. This intricate synaptic arrangement, coupled with such subregional targeting, provides a sophisticated means by which the LH can fine-tune pain transmission.”.

*2. The GABA immunofluorescence images in Fig. 1E and 1F are of poor quality, failing to distinguish background from specific GABA staining. Higher-quality images are recommended.*

**Response:**  We appreciate the reviewer for this recommendation. As suggested, we replaced the representative images in Fig 1D, 1E, and 1F with higher-quality versions that provide clearer visualization and better signal-to-noise ratio. Accordingly, we have updated the statistical results in panels Fig. 1G, 1H, and 1I. We have confirmed that these refinements do not alter the results reported in the study.

*3. As shown in Fig. 1C, neurons in the LH that project to both the PVT and LPB are present. The function of these neurons (e.g., co-regulating pain and emotion) should be discussed in the Discussion section.*

**Response:** We have updated the Discussion to address the neurons dually projecting to the PVT and LPB. We now discuss how this subpopulation may link the multi-functional LH-PVT pathway with the sensory-specific LH-LPB pathway to coordinate pain and emotion (see Discussion, lines 741-745).

In the Discussion section, we wrote: “It is worth noting that a small fraction of LH GABAergic neurons (~7.5%) sends collateral projections to both the PVT and LPB. These dual-projecting neurons may provide a structural basis for the co-regulation of pain and emotion. By simultaneously engaging both targets, this subpopulation could synchronize physiological nociception with negative affective states, ensuring a unified response to noxious stimuli.”

*4. No neuronal somata with viral expression were observed in Fig. 2C, which is inappropriate and should be corrected.*

**Response**: We apologize for the lack of clarity in Fig. 2C of the previous version. We have now replaced it with an image that clearly shows the viral expression in the neuronal somata within the LH.

*5. No cannula traces are found in the PVT (Figs. 2B, 2C, 5B) and LPB (Figs. 3B, 5I) regions; the reasons should be explained.*

**Response**: We appreciate the reviewer's comment. In the original figures, we selected representative images that prioritized the complete visualization of viral expression and fluorescence signal quality, as extensive tissue damage from cannula tracks can interfere with high-resolution imaging and fluorescence quantification. To provide clear evidence of our histological verification, we have now included representative histological images with visible optical fiber tracks for both PVT and LPB in Supplementary Fig 3, 4, 6 and 8. We have also updated the Methods section to state (lines 839-849) that the placement of each fiber was histologically verified post-hoc, and only data from mice with correctly targeted implants were included in the final analysis.

*6.* *Previous studies show that enhanced activity of PVT glutamatergic neurons promotes anxiety (Di Li, Neuron, 2025; Shao-Hua Liang, J Neurosci, 2020; Qian-Qian Tang, Neuropsychopharmacology, 2024). However, this study finds that enhanced inhibitory inputs to these neurons also promote anxiety; the reasons for this contradiction should be analyzed in the Discussion.*

**Response:** We appreciate the reviewer’s suggestion regarding the contradiction between our results and the literature. We have now addressed this in the Discussion section (Lines 697-715). We propose that the pro-anxiety effect of LH GABAergic inputs may be attributed to functional heterogeneity of PVT subpopulations (such as aPVT vs. pPVT and their specific input-output motifs) and the unique electrophysiological properties of thalamic neurons. Furthermore, our study targets a specific LH-originated circuit rather than non-specific modulation of the entire nucleus. This specific recruitment of PVT sub-circuits may differently impact the E/I balance compared to broad glutamatergic activation.

In the Discussion section, we wrote: “The observation that inhibiting the LH-PVT GABAergic projection produces analgesic and anxiolytic effects is contrast towith the reported function of the PVT. Literature reports that activation of PVT glutamatergic neurons and their outputs to the vmPFC or BLA is associated with increased anxiety and negative affect [12, 23, 24]. This contradiction likely reflects the unique electrophysiological properties of PVT neurons. Sustained GABAergic inhibition hyperpolarizes and silences these neurons, meanwhile de-inactivates T-type calcium channels, which promotes rebound burst firing [25]. The latter may potently drive downstream anxiety-related nuclei. In fact, the PVT is a diverse nucleus where distinct neuronal populations exert opposite effects on emotional processing [12, 26]. For instance, activation of PVT neurons receiving oxytocinergic inputs from the PVN or projections from the preBötC relieve anxiety [27, 28]. In this study, we modulated PVT neurons inhibited by LH GABAergic neurons, which mayprobably resulting in different outcomes from modulation of PVT neurons in general. Such precise modulation likely influences specific subpopulations or altersing the excitatory/inhibitory balance within distinct circuit motifs [29]. Collectively, these results demonstrate that the LH-PVT pathway recruits complex mechanisms—potentially involving firing-mode switching or disinhibition—that significantly contribute to the growing understanding of the PVT’s functional complexity in pain and emotional processing.”.

*7. Glutamatergic (VGlut2-expressing) and GABAergic (GAD67-expressing) neurons are generally independent subtypes. However, some neurons in Fig. 6B co-express GAD67-mCherry and Vglut2, and the reason should be explained.*

**Response:** Regarding the reviewer's observation, we have replaced the previous image with a better one in revision. The perceived co-localization in the original figure was an optical artifact primarily due to its limited resolution and the high density of neurons in the PB, where strong fluorescence signals from adjacent cell bodies can appear to overlap in the merged image. Our updated images in Fig. 6B more clearly demonstrate that the GAD67-mCherry and Vglut2 signals represent distinct neuronal populations, consistent with our quantitative analysis.

*8. The Vglut2 staining in Fig. 6E is poor, making it hard to identify positive neurons; replace with appropriate images. Additionally, there is an inconsistency in the anatomical levels of LPB presented between the laser on and laser off groups, which may affect the reliability of the experimental results. A modification is advised.*

**Response:** We apologize for the quality of images in Fig. 6E in the previous version. As suggested, we have repeated the experiments in additional mice and replaced Fig. 6B and 6G with better images where Vglut2+ neurons are clearly identifiable. The corresponding quantification has also been updated to include the new data.

Regarding the inconsistency in the anatomical levels of the LPB between images from “Light on” and “Light off” groups in Fig 6G, we replaced them with new images with matched anatomical levels (Bregma approx. -5.2 mm).

*9. No control virus is set in Fig. 8; relevant control experiments should be supplemented.*

**Response:** We agree with the reviewer on this point. We performed additional control experiments using AAV-EF1α-DIO-mCherry (instead of hM3Dq) in the LPB of Vgat-Cre mice, while still expressing ChR2 in the LH GABAergic neurons. Our results showed that in the absence of hM3Dq, CNO administration failed to block the effects of LH-LPB projection stimulation on pain behavior in either naive mice or SNI mice (Fig 8E-F, I-J). The Results section (Lines 653-657, 664-665) have been updated accordingly.

**Reviewer #2**

*In this manuscript, Sun et al. presented data that characterize the functional divergence of two parallel GABAergic projections from the lateral hypothalamus (LH) to the paraventricular nucleus of the thalamus (PVT) and the lateral parabrachial nucleus (LPB). Using a combination of retrograde labeling, fiber photometry, and optogenetic manipulations, the authors demonstrate that while both pathways encode salient and affective information, they serve distinct roles: the LH-PVT circuit selectively modulates anxiety-like behaviors, whereas the LH-LPB circuit primarily regulates nociception and its associated affective dimensions. Specifically, the study suggests that LH GABAergic inputs to the LPB enhance the excitability of glutamatergic neurons during noxious challenges. While the anatomical and functional segregation of these circuits is of high interest to the field of neurobiology and pain research, the impact of the findings is currently limited by significant variability in the control groups, a lack of clarity regarding certain viral and experimental strategies, and the need for more rigorous controls/discussion establishing the specific relevance of the LH-LPB pathway on LPB glutamatergic neurons in the absence of noxious stimuli.*

***Major:***

*1. There is some concern regarding the apparent differences observed in the eYFP groups in Figure 2. For example, there is a clear difference in the OFT eYFP mouse behavior in the 2F1/G1 and 2K1/L1. Presumably, these are the "same" mice as they are injected with the same virus, the only difference is the light wavelength used. If you were to use the eYFP data in Figure 2K1 in Figure 2F1 (or vice versa), the difference looks to be minimal. This is a bit concerning.*

**Response:** We appreciate the reviewer’s careful observation. We would like to clarify that the ChR2 (Figs. 2F–G) and NpHR (Figs. 2K–L) experiments were performed using independent cohorts of mice at different time points.

Regarding the OFT, we agree that the previous representative track plots did not optimally reflect the consistency between groups. We have now replaced the track plots in Fig 2F1 and 2K1 with images that better represent the average behavioral patterns of each cohort. These updated images, combined with the quantified statistical data (total distance and velocity in 2F4, 2F5 and 2K4, 2K5), demonstrate that the locomotor activity and exploratory behavior of eYFP mice remained consistent across different cohorts and light conditions.

Regarding the EPM, we acknowledge the baseline differences in open arm duration between the eYFP groups in Figs 2G3 and 2L3. Such variations in baseline anxiety levels are well-recognized in behavioral neuroscience and can be attributed to different animal batches, seasonal variations, or subtle environmental fluctuations. To maintain scientific rigor, we employed a concurrent control for each independent cohort. The behavioral effects were evaluated by comparing the experimental group (ChR2 or NpHR) directly with its own time-matched control group. Despite these baseline variations, the relative changes induced by optogenetic modulation were consistent, supporting our conclusion that the LH-PVT pathway bidirectionally regulates anxiety-like behavior.

*2. It is unclear why the data in 2H3 and 2M3 are so different. The heat maps in 2H1 and 2M1 do not indicate a significant difference in behavior between the two cohorts. The Methods explanation indicates a 20 minute pre-conditioning session and the assumption is this the pre-test data for 2H1 and 2M1. However, the average time for the NpHR group (~400 seconds or 6.5 minutes) in the light-side pre-test was evidently lower than the ChR2 group (~750 seconds, 12.5 minutes). The assumption is that this was deliberate, but this needs to be clarified.*

**Response:** We thank the reviewer for this comment. The difference in Pre-test baselines between the ChR2 and NpHR groups resulted from a deliberate counterbalanced experimental design. Specifically, for the ChR2 group (expected pro-nociceptive/ aversive effect), we paired light stimulation with the initially preferred side to detect a potential decrease. Conversely, for the NpHR group (expected anti-nociceptive/rewarding effect), light was paired with the initially non-preferred side to observe a potential increase. This approach was employed to avoid ceiling or floor effects and ensures that the observed behavioral shifts (Post-test vs. Pre-test) reflect the functional role of the LH-LPB circuit in pain modulation.

We have also clarified this experimental design in the revised Methods section (Lines 893-897).

*3. Nowhere is there clarification on whether correct placement of optical fibers was confirmed following behavioral experiments and how this was done. It looks as if it is shown in select images in Figures 6 and 7, but that is it. In fact, it is unclear what the fiber outline in Figure 6B represents as the schematic in Figure 6A does not include a fiber.*

**Response:** We agree with the reviewer that histological confirmation is crucial. To maintain the visual quality of the representative immunofluorescence images in the figures, we provided representative bright field images showing the fiber tracks in S3, 4, 6 and 8 Fig. These images illustrate how we verified the placement of optical fiber tips. Additionally, we updated the Methods section (Lines 839-849) to clarify that only mice with correctly placed fibers and verified viral expression were included in the final analysis. For Figures 6 and 7, we replaced the previous images with higher-resolution versions to ensure that the schematics and representative images are consistent in their presentation.

*4. There looks to be a trending reduction in open arm duration upon LH-LPB activation by ChR2 (Figure 3F3). Power analyses would be informative.*

**Response:** We appreciate the reviewer's observation. We performed a post-hoc power analysis on the open arm duration (*P* = 0.11, *n* = 7 per group). The achieved power is 0.378. The relatively low power is primarily due to the inherent variability of behavior in the eYFP control group and the modest sample size. However, given the lack of change in open arm entries (*P* = 0.70), and consistent results in the Open Field Test (Fig. 3E)-showed no significant changes, we maintained a conservative interpretation. We conclude that while a trend exists, this circuit does not exert a robust effect on anxiety-like behavior,

*5. It is unclear why OFT and EPM behaviors were not performed in a before (light-off) and after (light-on) configuration. There is considerable variability for instance in the NpHR data shown in Figure 4D2, which may be relevant if compared within animal to behavior with no light.*

**Response:** We appreciate the reviewer’s suggestion. To maintain the validity of anxiety assessments, we used a between-subjects design rather than a before/after configuration. Since OFT and EPM rely heavily on the initial environmental novelty, repeated testing (within-animal) would lead to habituation artifacts, potentially masking the circuit's effects. Testing naive mice in a single session is a widely accepted approach to ensure that the observed behaviors reflect innate anxiety rather than learned safety or habituation.

*6. More explanation of the viral strategies used for data shown in Figure 6 is needed either in Methods or in the Results section describing Figure 6.*

**Response:** We have updated the Results section to clearly describe the viral strategy used in Figure 6. We now specify that the AAV-DIO-WGA-Flp and AAV-fDIO-eYFP system was used for anterograde trans-synaptic labeling, allowing us to identify and quantify the specific postsynaptic targets (GABAergic vs. glutamatergic neurons) of LH GABAergic projections in the LPB.

In the Results section, we wrote: “To elucidate the direct cellular targets of LH GABAergic projections within the LPB, we employed an anterograde trans-synaptic labeling strategy. Specifically, we injected AAV-hSyn-DIO-WGA-Flp into the LH of Vgat-Cre mice (Fig 6A). This virus expresses a WGA-Flp fusion protein in LH-GABAergic neurons, which is then transported trans-synaptically to label postsynaptic neurons. Concurrently, a Flp-dependent reporter (AAV-hSyn-fDIO-eYFP) was injected into the LPB to selectively label LPB neurons receiving direct synaptic inputs from LH GABAergic neurons. To determine the identity of these neurons, AAV-GAD67-mCherry was co-injected to label GABAergic cells, and Vglut2-antibody staining was used to label glutamatergic neurons.”.

*7. In addition, it is not specified how many mice/slices were used to generate data in Figure 6C.*

**Response:** We updated Figure 6C and the corresponding figure legend to specify the sample size. The quantification was performed on 146 eYFP-positive neurons from 5 mice.

*8. It is stated "Successful viral expression and fiber implantation were confirmed by representative fluorescence image (Figure 7B)", but it is unclear why the authors did show red axon haze in LPB instead of just soma expression in LH.*

**Response:** We revised Figure 7B to show both the viral expression in the LH (soma) and the resulting axonal projections in the LPB. This provides clearer evidence of both the successful injection site and the specific innervation of the target region.

*9. The authors state "LH GABAergic input to LPB glutamatergic neurons enhances their excitability during noxious mechanical and thermal challenges", which looks to be true, but the relevance of this statement is unclear as they do not show if the activation LH GABAergic input does anything to LPB glutamatergic neuron activity in the absence of a noxious challenge. If it does, then the impact/relevance of this pathway in pain is unclear. While I know there are likely limitations of virus availability that would allow for 633 nm induced INHIBITION of LH GABAerigic, that is the more relevant experiment showing that blocking activation of LH GABAergic inputs eliminates the activation of LPB glutamatergic neurons in response to noxious input.*

**Response:** We appreciate the reviewer for this insightful comment. To clarify the functional relevance, our fiber photometry data (S6E-S6G Fig) were recorded without external noxious stimulation. As shown, activating LH-GABAergic terminals (633 nm) is sufficient to immediately increase calcium activity in LPB glutamatergic neurons (CaMKII+) (S6E Fig, middle panel), while inhibiting GABAergic neurons (Vgat+) (S6E Fig, left panel). This evidence establishes that the LH-LPB GABAergic pathway is sufficient to drive activity of LPB neurons. Furthermore, our NpHR experiments (Fig 6D-H) show that inhibiting LH-LPB GABAergic pathway reduces c-Fos expression in LPB glutamatergic neurons following SNI, confirming its necessity during pain processing.

*10. A summary schematic would be helpful to communicate the proposed circuit the authors describe based on the data.*

**Response:** We thank the reviewer for this suggestion. We have added a summary schematic (S9 Fig) to illustrate the proposed circuit logic. Our data indicate that the LH-GABAergic system modulates pain and anxiety via two distinct non-overlapping neural populations: one directly inhibiting the PVT to regulate anxiety and pain, and the other enhancing LPB excitatory output through a disinhibition mechanism (inhibiting LPB-GABAergic interneurons) to modulate pain.

***Minor:***

*1.* *LPB and LH text labelling is not clear in Figure 1A and could be repositioned for clarity.*

**Response:** We thank the reviewer for this suggestion. We have repositioned the "LPB" and "LH" labels to ensure that they do not overlap with the colored regions.

*2. There seems to be a small disparity in font for "PIC" in Figure 1K.*

**Response:** We thank the reviewer for pointing out this mistake. We have updated the abbreviation of picrotoxin from "PIC" to "PTX" in Figures 1K-Q to align with standard nomenclature. Additionally, we standardized the font style and size for "PTX" to ensure consistency throughout all panels.

*3. The statement "PVT-projecting neurons were more than LPB-projecting neurons" needs to be grammatically updated. Something like "**labeling of PVT-projecting neurons was more prevalent than that of LPB-projecting neurons."*

**Response:** We have revised the sentence as suggested. It now reads: "Quantitative analysis demonstrated that the labeling of PVT-projecting neurons was more prevalent than that of LPB-projecting neurons” (Lines 113-114).

*4. Anatomical directionality/orientation (i.e. medial/lateral, dorsal/ventral) in images would be informative.*

**Response:** We agree with the reviewer for this comment. We added orientations (D/M for dorsal/medial or D/L for dorsal/lateral) to all representative images in Figures.

*5. In the Results section describing Figure 7, it seems that this statement "We first investigated the effect of LH GABAergic neuron activation on LPB GABAergic neurons" should read LPB glutamatergic neurons.*

**Response:** We apologize for this mistake. The sentence was corrected (line 536).

*6. Typically glutamatergic neurons are represented as triangles while GABAergic are represented as circles. While this is primarily the case in the cortex, it still seems somewhat backwards in Figure 7.*

**Response:** We appreciate the reviewer for correcting us about the diagrams. We have updated the schematic in Figure 7, now representing glutamatergic neurons with triangles and GABAergic neurons with circles.

*7. It seems the authors are labeling the outline of a fiber tract in Figure 7B, but this is not clear.*

**Response:** We have added corresponding bright-field (BF) images in the Supplementary Information (Supplementary Figures 3, 4, 6 and 8). These BF images clearly show the fiber tracts.

**Reviewer #3**

*In the manuscript entitled "Two parallel LH GABAergic projections differentially modulate pain and anxiety", Sun et al. report that GABAergic neurons in the lateral hypothalamus (LH) send projections to both the periventricular nucleus of the thalamus (PVT) and the lateral parabrachial nucleus (LPB), and that these two projections play different roles in modulating pain and emotion. The authors first showed that PVT- and LPB-projecting LH GABAergic neurons are largely separate populations. The authors then demonstrated that the LH-PVT GABAergic projection regulates both pain and anxiety, whereas the LH-LPB GABAergic projection modulates pain but not anxiety. The authors also revealed that LH GABAergic neurons exert both direct inhibitory and indirect disinhibitory effects on LPB glutamatergic neurons, with the indirect disinhibitory effects predominating in pain modulation. This study is very interesting and advances our understanding of circuits originating from LH GABAergic neurons and their roles in modulating pain and anxiety. However, some concerns still need to be addressed to improve the quality of this manuscript.*

***Major Points:***

*1.In the figure legend of Fig. 1D-F, the color of GABA+ neurons is magenta, not the indicated color (blue). In some images, white and cyan arrowheads appear, but the authors only mention that "white arrowheads indicate colocalized neurons." In fact, white arrowheads indicate colocalization of mCherry with GABA, and cyan arrowheads indicate colocalization of eGFP with GABA. This should be corrected. Additionally, the abbreviation for picrotoxin in Fig. 1K-Q should be "PTX" rather than "PIC."*

**Response:** We apologize for these inaccuracies in the figure and legend. We have thoroughly revised Figure 1 and its corresponding legend as follows:

We corrected the color coding in the legend for Figure 1C-F to correctly reflect the data. In the revised Figure 1, we explicitly defined the symbols: open arrowheads denote neurons co-projecting to both the PVT and LPB; arrows now denote the colocalization of LPB-projecting neurons with GABA; and filled arrowheads denote the colocalization of PVT-projecting neurons with GABA. As suggested, we replaced "PIC" with "PTX" for the abbreviation of picrotoxin throughout Figures 1K-Q and the corresponding texts in the Results section.

*2.In Fig. 2, the authors should add a caption noting that the behavioral data are from SNI mice. In Fig. 2H1 and 2M1, the indicators (blue and orange triangles) for the light-pairing side are too small to be noticed and should be enlarged. Furthermore, the paradigm of optogenetic stimulation and inhibition during the conditioning sessions for CPP should be explained in the legend or the Results section.*

**Response:** We revised the legend of Figure 2 to clarify the CPP paradigm (Lines 218-221). Specifically, we noted that the data were collected from SNI mice and provided a detailed description of the conditioning sessions (30-min morning/afternoon sessions with fixed-time optogenetic manipulation). For consistency, we also ensured that all other figure legends containing CPP data either include this description or refer to the paradigm in Figure 2. We also enlarged the triangles in Fig. 2H1 and 2M1 as suggested.

*3.Fig. 6E-G shows that inhibition of the LH-LPB GABAergic projection reduced the number of c-Fos+ neurons in the LPB. In my opinion, the percentage of LPB glutamatergic neurons activated is too high for naïve mice. The authors should clarify whether these images were from naïve or SNI mice.*

**Response:** We thank the reviewer for this point. We clarified that the images and quantification in Figure 6D-H were indeed obtained from SNI mice, which explains the high level of LPB activation. We added this information to the legend of Figure 6 (Lines 489) and the corresponding texts in the Results section (Lines 513-524).

*4.Combining Fig. 7 with Fig. S3,* *the effects of LH-LPB GABAergic terminal stimulation are weaker in SNI mice than in naïve mice. The authors should comment on this in the Discussion. Could this be related to hyperactivity of LH GABAergic neurons in SNI mice? That is, stimulation of the LH-LPB projection exerts diminished effects on LPB circuitry in SNI mice due to higher baseline activity. Examining c-Fos+ neurons in LPB-projecting LH neurons in sham versus SNI mice would convincingly address this issue.*

**Response:** We agree with the reviewer that the modulation might be less pronounced in SNI mice. To investigate the potential mechanism, we performed c-Fos-staining on LPB-projecting LH GABAergic neurons 3 weeks after SNI or sham surgery. Our data confirmed a significant increase in c-Fos-expression in SNI mice (see added supplementary Figure 5), supporting the reviewer’s hypothesis of baseline hyperactivity in LPB in SNI mice.

To ensure comparable pain intensity, we used suprathreshold stimuli adjusted to each group’s respective withdrawal thresholds (2.0 g for Naïve vs. 0.4g for SNI). Given that the stimulation still significantly enhanced GCaMP6 signal in SNI mice, we conclude that the LH-LPB projection remains as a potent modulator of chronic pain. We added a corresponding section in the Discussion to address this point (Lines 777-783).

*5.In Fig. 8, the authors should provide an image showing whether ChR2-eYFP is expressed in the LH without spreading to other nuclei.*

**Response:** We added representative images in the revised Figure 8 to demonstrate the specificity of ChR2-eYFP expression in the LH. In addition, we provided images showing ChR2-labeled axonal terminals within the LPB, and confirmed that the optic fiber tracks were accurately positioned above the target region (now shown in Fig 8 and S8 Fig).

*6.Although LH-LPB GABAergic neurons responded to both pain and emotional stimulation (Fig. 5H-N2), they regulated only pain thresholds (Figs. 3, 4). The authors should address this in the Discussion section.*

**Response:** We agree with the reviewer that this is a critical point. We added a paragraph in the Discussion (Lines 726-740) to address this functional dissociation.

We Wrote: “In stark contrast to the LH-PVT GABAergic pathway, this study revealed that the LH-LPB GABAergic projection primarily and unilaterally regulates nociception across both physiological and neuropathic pain states, with minimal influence on general affective or rewarding/aversive behaviors. While the LPB is a well-established core processing nociceptive, aversive, and emotional information [9, 30-32], our calcium imaging data showed multi-modal responses in LH-LPB GABAergic terminals, while modulation of these terminals only altered pain thresholds, but not emotional states. Recent studies have demonstrated that distinct LPB neuronal populations, defined by their genetic markers and projection targets, separately encode the sensory and affective components of pain [4, 5, 8, 33]. The LH-LPB GABAergic input may selectively modulate the sub-circuits dedicated to nociceptive signaling, while the broader affective-aversive states may require activation of LPB neurons projecting to nuclei such as the amygdala or BNST [8]. Our findings thus highlight that under the specific input of the LH, the LPB's GABAergic circuitry may be selectively harnessed for the precise modulation of pain sensation, rather than engaging emotion-related functions.”.

***Minor Points:***

*1.The first sentence of the 2nd paragraph in the 2nd part of Results: "Following these surgical procedures, mice underwent spared nerve injury of the sciatic nerve (SNI) surgery" should read as "Following these surgical procedures, mice underwent spared nerve injury (SNI) or sham surgery." This revision is necessary because, in Figure S1, the authors compared pain- and emotion-like behaviors between sham and SNI mice.*

**Response:** We thank the reviewer for this correction. The texts were revised to include sham surgery as suggested.

*2.In Figure S2, the authors should add captions above the respective panels to indicate that bidirectional optogenetic modulation was applied to the LH-PVT glutamatergic projection. Specifically, "Optogenetic activation of the LH-PVT projection" should be placed above panels A1-C1, and "Optogenetic inhibition of the LH-PVT projection" above panels F1-H1.*

**Response**: We thank the reviewer for this correction. The captions were updated accordingly.

*3.The caption at the top of Figure S3 should be corrected to: "GCaMP6 in LPB Glu Neurons in SNI mice."*

**Response**: We thank the reviewer for this correction. The caption was corrected accordingly.

*4.In Figs. 3B, 4B, 5B, 5I, 7G, and 8B, the position of the optical fiber implant, although discernible, should be clearly marked.*

**Response**: We added bright-field images showing the fiber tracks in S3, 4, 6 and 8 Fig, and clearly marked the fiber positions in the main figures.

*5.In Figures 4E1, 4H1, and 4I1, the panels show moving tracks, not "heatmaps." The labels should be corrected accordingly.*

**Response**: We thank the reviewer for this correction. The labels were changed from "heatmaps" to "moving tracks”.
