# Supplementary material for: Two parallel lateral hypothalamic GABAergic projections differentially modulate pain and anxiety in mice
Source: PLoS Biol. 2026 Nov 5;24(11):e3004096. doi: 10.1371/journal.pbio.3004096 (PMC13739175; doi:10.1371/journal.pbio.3004096)
Supplement: Peer review history

# Editor report: Decision Letter 0

**DOI:** 10.1371/journal.pbio.3004096.r001

**Authors:** Taylor Hart, PhD

3 Apr 2026

Dear Dr Zhou,

Thank you for submitting your manuscript entitled "Two parallel LH GABAergic projections differentially modulate pain and anxiety" for consideration as a Research Article by PLOS Biology.

Your manuscript has now been evaluated by the PLOS Biology editorial staff, as well as by an academic editor with relevant expertise, and I am writing to let you know that we would like to send your submission out for external peer review.

However, before we can send your manuscript to reviewers, we need you to complete your submission by providing the metadata that is required for full assessment. To this end, please login to Editorial Manager where you will find the paper in the 'Submissions Needing Revisions' folder on your homepage. Please click 'Revise Submission' from the Action Links and complete all additional questions in the submission questionnaire.

Once your full submission is complete, your paper will undergo a series of checks in preparation for peer review. After your manuscript has passed the checks it will be sent out for review. To provide the metadata for your submission, please Login to Editorial Manager (https://www.editorialmanager.com/pbiology) within two working days, i.e. by Apr 05 2026 11:59PM.

If your manuscript has been previously peer-reviewed at another journal, PLOS Biology is willing to work with those reviews in order to avoid re-starting the process. Submission of the previous reviews is entirely optional and our ability to use them effectively will depend on the willingness of the previous journal to confirm the content of the reports and share the reviewer identities. Please note that we reserve the right to invite additional reviewers if we consider that additional/independent reviewers are needed, although we aim to avoid this as far as possible. In our experience, working with previous reviews does save time.

If you would like us to consider previous reviewer reports, please edit your cover letter to let us know and include the name of the journal where the work was previously considered and the manuscript ID it was given. In addition, please upload a response to the reviews as a 'Prior Peer Review' file type, which should include the reports in full and a point-by-point reply detailing how you have or plan to address the reviewers' concerns.

During the process of completing your manuscript submission, you will be invited to opt-in to posting your pre-review manuscript as a bioRxiv preprint. Visit http://journals.plos.org/plosbiology/s/preprints for full details. If you consent to posting your current manuscript as a preprint, please upload a single Preprint PDF.

Feel free to email us at plosbiology@plos.org if you have any queries relating to your submission.

Kind regards,

Taylor

Taylor Hart, PhD,

Associate Editor

PLOS Biology

thart@plos.org

# Peer review: Decision Letter 1

**DOI:** 10.1371/journal.pbio.3004096.r002

**Authors:** Taylor Hart, PhD

15 May 2026

Dear Dr Zhou,

Thank you for your patience while your manuscript "Two parallel LH GABAergic projections differentially modulate pain and anxiety" was peer-reviewed at PLOS Biology. It has now been evaluated by the PLOS Biology editors, an Academic Editor with relevant expertise, and by several independent reviewers.

In light of the reviews, which you will find at the end of this email, we would like to invite you to revise the work to thoroughly address the reviewers' reports.

As you will see, the reviewers expressed interest in the study question and the findings, but also raised a number of important concerns that would need to be addressed. The main issues were related to internal inconsistencies, variability in some of the controls, insufficient documentation of viral expression and fiber placement, and image quality / annotation. In your revision, you should fully address these concerns and provide a thorough response to all of the reviewers' points. In particular, the mechanistic experiments would need clearer controls or stronger justifications to support the proposed disinhibitory mechanism; regarding the point from Reviewer 1 about subregional specificity: the broad claims about PVT and LPB function would either need to be supported by subregional quantifications, or the claims would need to be softened and the limitations clearly discussed.

Given the extent of revision needed, we cannot make a decision about publication until we have seen the revised manuscript and your response to the reviewers' comments. Your revised manuscript is likely to be sent for further evaluation by all or a subset of the reviewers.

In addition to these revisions, you will need to complete some formatting changes, which you will receive in a follow up email. A member of our team will be in touch with a set of requests shortly.

We expect to receive your revised manuscript within 3 months. Please email us (plosbiology@plos.org) if you have any questions or concerns, or would like to request an extension.

At this stage, your manuscript remains formally under active consideration at our journal; please notify us by email if you do not intend to submit a revision so that we may withdraw it.

**IMPORTANT - SUBMITTING YOUR REVISION**

Your revisions should address the specific points made by each reviewer. Please submit the following files along with your revised manuscript:

1. A 'Response to Reviewers' file - this should detail your responses to the editorial requests, present a point-by-point response to all of the reviewers' comments, and indicate the changes made to the manuscript.

*NOTE: In your point-by-point response to the reviewers, please provide the full context of each review. Do not selectively quote paragraphs or sentences to reply to. The entire set of reviewer comments should be present in full and each specific point should be responded to individually, point by point.

You should also cite any additional relevant literature that has been published since the original submission and mention any additional citations in your response.

2. In addition to a clean copy of the manuscript, please also upload a 'track-changes' version of your manuscript that specifies the edits made. This should be uploaded as a "Revised Article with Changes Highlighted" file type.

*Re-submission Checklist*

When you are ready to resubmit your revised manuscript, please refer to this re-submission checklist: https://plos.io/Biology_Checklist

To submit a revised version of your manuscript, please go to https://www.editorialmanager.com/pbiology/ and log in as an Author. Click the link labelled 'Submissions Needing Revision' where you will find your submission record.

Please make sure to read the following important policies and guidelines while preparing your revision:

*Published Peer Review*

Please note while forming your response, if your article is accepted, you may have the opportunity to make the peer review history publicly available. The record will include editor decision letters (with reviews) and your responses to reviewer comments. If eligible, we will contact you to opt in or out. Please see here for more details:

https://blogs.plos.org/plos/2019/05/plos-journals-now-open-for-published-peer-review/

*PLOS Data Policy*

Please note that as a condition of publication PLOS' data policy (http://journals.plos.org/plosbiology/s/data-availability) requires that you make available all data used to draw the conclusions arrived at in your manuscript. If you have not already done so, you must include any data used in your manuscript either in appropriate repositories, within the body of the manuscript, or as supporting information (N.B. this includes any numerical values that were used to generate graphs, histograms etc.). For an example see here: http://www.plosbiology.org/article/info%3Adoi%2F10.1371%2Fjournal.pbio.1001908#s5

*Blot and Gel Data Policy*

We require the original, uncropped and minimally adjusted images supporting all blot and gel results reported in an article's figures or Supporting Information files. We will require these files before a manuscript can be accepted so please prepare them now, if you have not already uploaded them. Please carefully read our guidelines for how to prepare and upload this data: https://journals.plos.org/plosbiology/s/figures#loc-blot-and-gel-reporting-requirements

*Protocols deposition*

To enhance the reproducibility of your results, we recommend that if applicable you deposit your laboratory protocols in protocols.io, where a protocol can be assigned its own identifier (DOI) such that it can be cited independently in the future. Additionally, PLOS ONE offers an option for publishing peer-reviewed Lab Protocol articles, which describe protocols hosted on protocols.io. Read more information on sharing protocols at https://plos.org/protocols?utm_medium=editorial-email&utm_source=authorletters&utm_campaign=protocols

Thank you again for your submission to our journal. We hope that our editorial process has been constructive thus far, and we welcome your feedback at any time. Please don't hesitate to contact us if you have any questions or comments.

Sincerely,

Taylor

Taylor Hart, PhD,

Associate Editor

PLOS Biology

thart@plos.org

------------------------------------

REVIEWS:

Reviewer #1: This study investigates two parallel GABAergic projection pathways from the LH to the PVT and the LPB and their differential modulation of pain and anxiety. The research reveals two functionally segregated neural circuits, providing new insights into the mechanistic basis of pain-emotion comorbidity, demonstrating significant innovation. However, the manuscript could be further improved in terms of image quality and result justification. We encourage additional revisions to strengthen the overall quality and academic rigor of the study. Specific comments are provided below.

1.Previous studies have demonstrated that the regulation of pain and emotion by the PVT and LPB exhibits subregion-specific differences. Therefore, do the projections and functions of the LH to the PVT and LPB also exhibit subregional specificity? It is recommended to supplement this with relevant experiments for clarification.

2.The GABA immunofluorescence images in Fig. 1E and 1F are of poor quality, failing to distinguish background from specific GABA staining. Higher-quality images are recommended.

3.As shown in Fig. 1C, neurons in the LH that project to both the PVT and LPB are present. The function of these neurons (e.g., co-regulating pain and emotion) should be discussed in the Discussion section.

4.No neuronal somata with viral expression were observed in Fig. 2C, which is inappropriate and should be corrected.

5.No cannula traces are found in the PVT (Figs. 2B, 2C, 5B) and LPB (Figs. 3B, 5I) regions; the reasons should be explained.

6.Previous studies show that enhanced activity of PVT glutamatergic neurons promotes anxiety (Di Li, Neuron, 2025; Shao-Hua Liang, J Neurosci, 2020; Qian-Qian Tang, Neuropsychopharmacology, 2024). However, this study finds that enhanced inhibitory inputs to these neurons also promote anxiety; the reasons for this contradiction should be analyzed in the Discussion.

7. Glutamatergic (VGlut2-expressing) and GABAergic (GAD67-expressing) neurons are generally independent subtypes. However, some neurons in Fig. 6B co-express GAD67-mCherry and Vglut2, and the reason should be explained.

8.The Vglut2 staining in Fig. 6E is poor, making it hard to identify positive neurons; replace with appropriate images. Additionally, there is an inconsistency in the anatomical levels of LPB presented between the laser on and laser off groups, which may affect the reliability of the experimental results. A modification is advised.

9.No control virus is set in Fig. 8; relevant control experiments should be supplemented.

Reviewer #2 [Patrick L. Sheets]: In this manuscript, Sun et al. presented data that characterize the functional divergence of two parallel GABAergic projections from the lateral hypothalamus (LH) to the paraventricular nucleus of the thalamus (PVT) and the lateral parabrachial nucleus (LPB). Using a combination of retrograde labeling, fiber photometry, and optogenetic manipulations, the authors demonstrate that while both pathways encode salient and affective information, they serve distinct roles: the LH-PVT circuit selectively modulates anxiety-like behaviors, whereas the LH-LPB circuit primarily regulates nociception and its associated affective dimensions. Specifically, the study suggests that LH GABAergic inputs to the LPB enhance the excitability of glutamatergic neurons during noxious challenges. While the anatomical and functional segregation of these circuits is of high interest to the field of neurobiology and pain research, the impact of the findings is currently limited by significant variability in the control groups, a lack of clarity regarding certain viral and experimental strategies, and the need for more rigorous controls/discussion establishing the specific relevance of the LH-LPB pathway on LPB glutamatergic neurons in the absence of noxious stimuli.

Major:

1.There is some concern regarding the apparent differences observed in the eYFP groups in Figure 2. For example, there is a clear difference in the OFT eYFP mouse behavior in the 2F1/G1 and 2K1/L1. Presumably, these are the "same" mice as they are injected with the same virus, the only difference is the light wavelength used. If you were to use the eYFP data in Figure 2K1 in Figure 2F1 (or vice versa), the difference looks to be minimal. This is a bit concerning.

2.It is unclear why the data in 2H3 and 2M3 are so different. The heat maps in 2H1 and 2M1 do not indicate a significant difference in behavior between the two cohorts. The Methods explanation indicates a 20 minute pre-conditioning session and the assumption is this the pre-test data for 2H1 and 2M1. However, the average time for the NpHR group (~400 seconds or 6.5 minutes) in the light-side pre-test was evidently lower than the ChR2 group (~750 seconds, 12.5 minutes). The assumption is that this was deliberate, but this needs to be clarified.

3.Nowhere is there clarification on whether correct placement of optical fibers was confirmed following behavioral experiments and how this was done. It looks as if it is shown in select images in Figures 6 and 7, but that is it. In fact, it is unclear what the fiber outline in Figure 6B represents as the schematic in Figure 6A does not include a fiber.

4.There looks to be a trending reduction in open arm duration upon LH-LPB activation by ChR2 (Figure 3F3). Power analyses would be informative.

5.It is unclear why OFT and EPM behaviors were not performed in a before (light-off) and after (light-on) configuration. There is considerable variability for instance in the NpHR data shown in Figure 4D2, which may be relevant if compared within animal to behavior with no light.

6.More explanation of the viral strategies used for data shown in Figure 6 is needed either in Methods or in the Results section describing Figure 6.

7.In addition, it is not specified how many mice/slices were used to generate data in Figure 6C.

8.It is stated "Successful viral expression and fiber implantation were confirmed by representative fluorescence image (Figure 7B)", but it unclear why the authors did show red axon haze in LPB instead of just soma expression in LH.

9.The authors state "LH GABAergic input to LPB glutamatergic neurons enhances their excitability during noxious mechanical and thermal challenges", which looks to be true, but the relevance of this statement is unclear as they do not show if the activation LH GABAergic input does anything to LPB glutamatergic neuron activity in the absence of a noxious challenge. If it does, then the impact/relevance of this pathway in pain is unclear. While I know there are likely limitations of virus availability that would allow for 633 nm induced INHIBITION of LH GABAerigic, that is the more relevant experiment showing that blocking activation of LH GABAergic inputs eliminates the activation of LPB glutamatergic neurons in response to noxious input.

10.A summary schematic would be helpful to communicate the proposed circuit the authors describe based on the data.

Minor:

1.LPB and LH text labelling is not clear in Figure 1A and could be repositioned for clarity.

2.There seems to be a small disparity in font for "PIC" in Figure 1K.

3.The statement "PVT-projecting neurons were more than LPB-projecting neurons" needs to be grammatically updated. Something like "labeling of PVT-projecting neurons was more prevalent than that of LPB-projecting neurons."

4.Anatomical directionality/orientation (i.e. medial/lateral, dorsal/ventral) in images would be informative.

5.In the Results section describing Figure 7, it seems that this statement "We first investigated the effect of LH GABAergic neuron activation on LPB GABAergic neurons" should read LPB glutamatergic neurons.

6.Typically glutamatergic neurons are represented as triangles while GABAergic are represented as circles. While this is primarily the case in the cortex, it stills seems somewhat backwards in Figure 7.

7.It seems the authors are labeling the outline of a fiber tract in Figure 7B, but this is not clear.

Reviewer #3: In the manuscript entitled "Two parallel LH GABAergic projections differentially modulate pain and anxiety", Sun et al. report that GABAergic neurons in the lateral hypothalamus (LH) send projections to both the periventricular nucleus of the thalamus (PVT) and the lateral parabrachial nucleus (LPB), and that these two projections play different roles in modulating pain and emotion. The authors first showed that PVT- and LPB-projecting LH GABAergic neurons are largely separate populations. The authors then demonstrated that the LH-PVT GABAergic projection regulates both pain and anxiety, whereas the LH-LPB GABAergic projection modulates pain but not anxiety. The authors also revealed that LH GABAergic neurons exert both direct inhibitory and indirect disinhibitory effects on LPB glutamatergic neurons, with the indirect disinhibitory effects predominating in pain modulation. This study is very interesting and advances our understanding of circuits originating from LH GABAergic neurons and their roles in modulating pain and anxiety. However, some concerns still need to be addressed to improve the quality of this manuscript.

Major Points:

1.In the figure legend of Fig. 1D-F, the color of GABA+ neurons is magenta, not the indicated color (blue). In some images, white and cyan arrowheads appear, but the authors only mention that "white arrowheads indicate colocalized neurons." In fact, white arrowheads indicate colocalization of mCherry with GABA, and cyan arrowheads indicate colocalization of eGFP with GABA. This should be corrected. Additionally, the abbreviation for picrotoxin in Fig. 1K-Q should be "PTX" rather than "PIC."

2.In Fig. 2, the authors should add a caption noting that the behavioral data are from SNI mice. In Fig. 2H1 and 2M1, the indicators (blue and orange triangles) for the light-pairing side are too small to be noticed and should be enlarged. Furthermore, the paradigm of optogenetic stimulation and inhibition during the conditioning sessions for CPP should be explained in the legend or the Results section.

3.Fig. 6E-G shows that inhibition of the LH-LPB GABAergic projection reduced the number of c-Fos+ neurons in the LPB. In my opinion, the percentage of LPB glutamatergic neurons activated is too high for naïve mice. The authors should clarify whether these images were from naïve or SNI mice.

4.Combining Fig. 7 with Fig. S3, the effects of LH-LPB GABAergic terminal stimulation are weaker in SNI mice than in naïve mice. The authors should comment on this in the Discussion. Could this be related to hyperactivity of LH GABAergic neurons in SNI mice? That is, stimulation of the LH-LPB projection exerts diminished effects on LPB circuitry in SNI mice due to higher baseline activity. Examining c-Fos+ neurons in LPB-projecting LH neurons in sham versus SNI mice would convincingly address this issue.

5.In Fig. 8, the authors should provide an image showing whether ChR2-eYFP is expressed in the LH without spreading to other nuclei.

6.Although LH-LPB GABAergic neurons responded to both pain and emotional stimulation (Fig. 5H-N2), they regulated only pain thresholds (Figs. 3, 4). The authors should address this in the Discussion section.

Minor Points:

1.The first sentence of the 2nd paragraph in the 2nd part of Results: "Following these surgical procedures, mice underwent spared nerve injury of the sciatic nerve (SNI) surgery" should read as "Following these surgical procedures, mice underwent spared nerve injury (SNI) or sham surgery." This revision is necessary because, in Figure S1, the authors compared pain- and emotion-like behaviors between sham and SNI mice.

2.In Figure S2, the authors should add captions above the respective panels to indicate that bidirectional optogenetic modulation was applied to the LH-PVT glutamatergic projection. Specifically, "Optogenetic activation of the LH-PVT projection" should be placed above panels A1-C1, and "Optogenetic inhibition of the LH-PVT projection" above panels F1-H1.

3.The caption at the top of Figure S3 should be corrected to: "GCaMP6 in LPB Glu Neurons in SNI mice."

4.In Figs. 3B, 4B, 5B, 5I, 7G, and 8B, the position of the optical fiber implant, although discernible, should be clearly marked.

5.In Figures 4E1, 4H1, and 4I1, the panels show moving tracks, not "heatmaps." The labels should be corrected accordingly.

# Author comment: Author response to Decision Letter 2

**DOI:** 10.1371/journal.pbio.3004096.r003

1 Sep 2026

Attachment Submitted filename: Reviewers comments-2026-8-31.docx

# Peer review: Decision Letter 2

**DOI:** 10.1371/journal.pbio.3004096.r004

**Authors:** Taylor Hart, PhD

6 Oct 2026

Dear Dr Zhou,

Thank you for your patience while we considered your revised manuscript "Two parallel LH GABAergic projections differentially modulate pain and anxiety" for publication as a Research Article at PLOS Biology. This revised version of your manuscript has been evaluated by the PLOS Biology editors, the Academic Editor, and the original reviewers.

Based on the reviews, we are likely to accept this manuscript for publication. Please also make sure to address the following data and other policy-related requests.

IMPORTANT: Please ensure that your next revision addresses the following points:

--------------------

**Title:

Because of the journal's interest in accessibility and appropriate contextualization for our broad readership, we would like to tweak the paper's title. Is the following alternative version acceptable to you?

"Two parallel lateral hypothalamic GABAergic projections differentially modulate pain and anxiety in mice"

**Data:

Thank you for providing the underlying numerical data; this is very helpful and close to what we need. We would request that you make a few additional changes to S1 Data:

- Please provide numerical data for Fig 1LNQS

- Please check that the data are correct and labeled properly for Fig 3E2,E3 and Fig 5D2, as we think that labeling errors may be present.

**Code availability:

Per journal policy, if you have generated any custom code or scripts during the course of this investigation, we require that you make it available without restrictions. Please ensure that the code is sufficiently well documented and reusable, and that your Data Statement in the Editorial Manager submission system accurately describes where your code can be found. Please also ensure that you choose a license for your code and include a Readme file.

Please note that we cannot accept sole deposition of code in GitHub, as this could be changed after publication. However, you can archive this version of your publicly available GitHub code to Zenodo. Once you do this, it will generate a DOI number, which you will need to provide in the Data Accessibility Statement (you are welcome to also provide the GitHub access information). See the process for doing this here: https://docs.github.com/en/repositories/archiving-a-github-repository/referencing-and-citing-content

**Species in abstract:

Please note that per journal policy, the model system/species studied should be clearly stated in the abstract of your manuscript.

--------------------

As you address these items, please take this last chance to review your reference list to ensure that it is complete and correct. If you have cited papers that have been retracted, please include the rationale for doing so in the manuscript text, or remove these references and replace them with relevant current references. Any changes to the reference list should be mentioned in the cover letter that accompanies your revised manuscript.

In addition to these revisions, you may need to complete some formatting changes, which you will receive in a follow up email. A member of our team will be in touch with a set of requests shortly. If you do not receive a separate email within a few days, please assume that checks have been completed, and no additional changes are required.

We expect to receive your revised manuscript within two weeks.

To submit your revision, please go to https://www.editorialmanager.com/pbiology/ and log in as an Author. Click the link labelled 'Submissions Needing Revision' to find your submission record. Your revised submission must include the following:

- a cover letter that should detail your responses to any editorial requests, if applicable, and whether changes have been made to the reference list

- a Response to Reviewers file that provides a detailed response to the reviewers' comments (if applicable, if not applicable please do not delete your existing 'Response to Reviewers' file.)

- a track-changes file indicating any changes that you have made to the manuscript.

NOTE: If Supporting Information files are included with your article, note that these are not copyedited and will be published as they are submitted. Please ensure that these files are legible and of high quality (at least 300 dpi) in an easily accessible file format. For this reason, please be aware that any references listed in an SI file will not be indexed. For more information, see our Supporting Information guidelines:

https://journals.plos.org/plosbiology/s/supporting-information

*Published Peer Review History*

Please note that you may have the opportunity to make the peer review history publicly available. The record will include editor decision letters (with reviews) and your responses to reviewer comments. If eligible, we will contact you to opt in or out. Please see here for more details:

https://plos.org/published-peer-review-history/

*Press*

Should you, your institution's press office or the journal office choose to press release your paper, please ensure you have opted out of Early Article Posting on the submission form. We ask that you notify us as soon as possible if you or your institution is planning to press release the article.

*Protocols deposition*

To enhance the reproducibility of your results, we recommend that if applicable you deposit your laboratory protocols in protocols.io, where a protocol can be assigned its own identifier (DOI) such that it can be cited independently in the future. Additionally, PLOS ONE offers an option for publishing peer-reviewed Lab Protocol articles, which describe protocols hosted on protocols.io. Read more information on sharing protocols at https://plos.org/protocols?utm_medium=editorial-email&utm_source=authorletters&utm_campaign=protocols

Please do not hesitate to contact me should you have any questions.

Sincerely,

Taylor

Taylor Hart, PhD,

Associate Editor

thart@plos.org

PLOS Biology

Reviewer remarks:

Reviewer #1: The MS has been significantly improved. I have no additional comments.

Reviewer #2: The authors have done an adequate job addressing my concerns.

I have no further comments that need to be addressed.

Reviewer #3: The authors have constructively addressed my concerns.

# Author comment: Author response to Decision Letter 3

**DOI:** 10.1371/journal.pbio.3004096.r005

12 Oct 2026

Attachment Submitted filename: Reviewers_comments-2026-8-31_auresp_3.docx

# Editor report: Decision Letter 3

**DOI:** 10.1371/journal.pbio.3004096.r006

**Authors:** Taylor Hart, PhD

19 Oct 2026

Dear Dr Zhou,

Thank you for the submission of your revised Research Article "Two parallel lateral hypothalamic GABAergic projections differentially modulate pain and anxiety in mice" for publication in PLOS Biology. On behalf of my colleagues and the Academic Editor, Guang Yang, I am pleased to say that we can in principle accept your manuscript for publication, provided you address any remaining formatting and reporting issues. These will be detailed in an email you should receive within 2-3 business days from our colleagues in the journal operations team; no action is required from you until then. Please note that we will not be able to formally accept your manuscript and schedule it for publication until you have completed any requested changes.

Please take a minute to log into Editorial Manager at http://www.editorialmanager.com/pbiology/, click the "Update My Information" link at the top of the page, and update your user information to ensure an efficient production process.

PRESS

We frequently collaborate with press offices. If your institution or institutions have a press office, please notify them about your upcoming paper at this point, to enable them to help maximise its impact. If the press office is planning to promote your findings, we would be grateful if they could coordinate with biologypress@plos.org. If you have previously opted in to the early version process, we ask that you notify us immediately of any press plans so that we may opt out on your behalf.

We also ask that you take this opportunity to read our Embargo Policy regarding the discussion, promotion and media coverage of work that is yet to be published by PLOS. As your manuscript is not yet published, it is bound by the conditions of our Embargo Policy. Please be aware that this policy is in place both to ensure that any press coverage of your article is fully substantiated and to provide a direct link between such coverage and the published work. For full details of our Embargo Policy, please visit http://www.plos.org/about/media-inquiries/embargo-policy/.

Thank you again for choosing PLOS Biology for publication and supporting Open Access publishing. We look forward to publishing your study.

Sincerely,

Taylor

Taylor Hart, PhD,

Associate Editor

PLOS Biology

thart@plos.org
